# Supplementary material for: Structural equation modeling to detect correlates of childhood vaccination: A moderated mediation analysis
Source: PLoS One. 2020 Oct 15;15(10):e0240749. doi: 10.1371/journal.pone.0240749 (PMC7561155; doi:10.1371/journal.pone.0240749)
Supplement: Attachment — Submitted filename: PONE-D-20-09473_R1_reviewer (comments, redacted).pdf [file pone.0240749.s002.pdf]

PLOS ONE

Structural equation modeling to detect correlates of childhood vaccination: a moderated mediation analysis

--Manuscript Draft--

**Manuscript Number:**	PONE-D-20-09473R1
**Article Type:**	Research Article
**Full Title:**	Structural equation modeling to detect correlates of childhood vaccination: a moderated mediation analysis
**Short Title:**	Structural equation modeling to detect correlates of childhood vaccination
**Corresponding Author:**	Abraham Degarege<br>University of Nebraska Medical Center<br>Omaha, UNITED STATES
**Keywords:**	Childhood vaccination, Structural equation model, Attitude, Parents, India
**Abstract:**	Abstract<br>Objectives<br>This study used a Health Belief Theory derived framework and Structural Equation Model to examine moderators, mediators and direct and indirect predictors of childhood vaccination.<br>Methods<br>A cross-sectional survey was conducted among a random sample of 1599 parents living in urban and rural areas of Mysore district, India. Applying two-stage probability proportionate-to-size sampling, adolescent girls attending 7th through 10th grades in public, private and religious schools located in the district were selected to take home a questionnaire to their parents. Parents were asked whether their children had received one dose of bacillus Calmette–Guérin; three doses of Diphtheria, Pertussis, Tetanus; three doses of oral polio vaccine; and one dose of measles-containing vaccine. In addition, parents were asked about their attitudes towards childhood vaccination.<br>Results<br>Out of the 1599 parents, 52.2% reported that their children had received all the routine vaccines (fully vaccinated); 42.7% reported their children had missed at least one routine vaccine, and 5.2% reported that their children had missed all routine vaccinations. Perceptions about the benefits/facilitators to childhood vaccination significantly predicted the full vaccination rate (standardized regression coefficient (β)=0.29) directly and mediated the effect of parental education (β=0.11) and employment (β= -0.06) on the rate of full vaccination. Parental education was significantly associated indirectly with higher rates of full vaccination (β=0.11). Parental employment was significantly associated indirectly with decreasing rates of full vaccination (β= -0.05). Area of residence moderated the role of religion (β= 0.24) and the 'number of children' in a family (β=0.33) on parental perceptions about barriers to childhood vaccination. The model to data fit was acceptable (Root Mean Square Error of Approximation = 0.02, 95% CI 0.018 to 0.023; Comparative Fit Index = 0.92; Tucker–Lewis Index = 0.91).<br>Conclusions<br>Full vaccination rate was relatively low among children in Mysore, especially among parents who were unsure about the benefits of routine vaccination and those with low educational levels. Interventions increasing awareness of the benefits of childhood vaccination that target rural parents with lower levels of education may help increase the rate of full childhood vaccination in India.
**Order of Authors:**	Abraham Degarege
	Karl Krupp
	Vijaya Srinivas
	Boubakari Ibrahimou
	Purnima Madhivanan
**Response to Reviewers:**	PONE-D-20-09473

Powered by Editorial Manager® and ProduXion Manager® from Aries Systems Corporation

Structural equation modeling to detect correlates of childhood vaccination: a moderated mediation analysis
PLOS ONE

Dear Dr. William Joe

Thank you for your comments and for providing us with the reviewers' comments as well. We thank also the reviewers for their careful review and constructive comments, which have helped to improve the manuscript. We have made changes to the manuscript based on reviewers' suggestions and describe these changes in the below paragraphs. We hope that you will find our responses acceptable and we look forward to your decision.

Editors

1. Please ensure that your manuscript meets PLOS ONE's style requirements, including those for file naming. The PLOS ONE style templates can be found at
https://journals.plos.org/plosone/s/file?id=wjVg/PLOSOne_formatting_sample_main_body.pdf
https://journals.plos.org/plosone/s/file?id=ba62/PLOSOne_formatting_sample_title_authors_affiliations.pdf
Response: We have checked the author guidelines for formatting of the manuscript and to assure the editor that it meets the requirements.
2. Please address the following:
- Please include additional information regarding the survey or questionnaire used in the study and ensure that you have provided sufficient details that others could replicate the analyses. For instance, if you developed a questionnaire as part of this study and it is not under a copyright more restrictive than CC-BY, please include a copy, in both the original language and English, as Supporting Information.
Response: We thank the editor for this suggestion. We have provided sufficient detail about the questionnaire used in this study that would enable others to replicate the analyses. In the manuscript, we have provided all the items and scales/response categories used to measure the outcome (childhood vaccination status) and exposure variables, including the latent (perceived benefits and perceived barriers) and measured variables (sociodemographic variables).

- Please refer to any sample size calculations performed prior to participant recruitment. If these were not performed please justify the reasons. Please refer to our statistical reporting guidelines for assistance
(https://journals.plos.org/plosone/s/submission-guidelines.#loc-statistical-reporting).
Response: This study was part of a survey implemented to examine HPV vaccine acceptance among parents of adolescent girls in India. The primary goal of the project was to examine factors associated with intention-to-vaccinate daughters with HPV vaccine. Assessing factors correlated with childhood vaccination was a secondary objective of the project. Therefore, the estimated sample size (n=1599) had enough power to test the hypotheses and answer the research question proposed related to both the objectives of the study.
3.Thank you for stating the following in the Financial Disclosure section:
'This study was funded by an Investigator Initiated Award from Merck & Co., Inc. PM received the fund (D43 TW010540). The funders had no role in study design, data collection and analysis, decision to publish, or preparation of the manuscript.'
We note that you received funding from a commercial source: Merck & Co., Inc.
a. Please provide an amended Competing Interests Statement that explicitly states this commercial funder, along with any other relevant declarations relating to employment, consultancy, patents, products in development, marketed products, etc.
Within this Competing Interests Statement, please confirm that this does not alter your adherence to all PLOS ONE policies on sharing data and materials by including the following statement: "This does not alter our adherence to PLOS ONE policies on sharing data and materials." (as detailed online in our guide for authors http://journals.plos.org/plosone/s/competing-interests). If there are restrictions on sharing of data and/or materials, please state these. Please note that we cannot proceed with consideration of your article until this information has been declared.
Response: We have provided the text below in the manuscript under the heading "funding"

Powered by Editorial Manager® and ProduXion Manager® from Aries Systems Corporation

"This study was funded by an Investigator-Initiated Award from Merck & Co., Inc. awarded to PM. KK was funded as a US Scholar by Fogarty International Center and National Heart Lung and Blood Institute, and National Institute of Neurological Disorders and Stroke and of the National Institutes of Health under Award Number D43 TW010540. PM was partially funded by grants from National Institutes for Health (D43-TW010540; R15-AI128714). The funder had no role in the study design, data collection, analysis, interpretation or publication of the manuscript."
We have also included the statement which reads, "This does not alter our adherence to PLOS ONE policies on sharing data and materials." under the heading “competing interest statement” and in the cover letter. However, I didn't see the financial disclosure and competing statements in the online submission and have not updated the text there. I would greatly appreciate if you can copy this text to the online submission as needed.

Thanks
b. Please include your amended Competing Interests Statement within your cover letter. We will change the online submission form on your behalf.
Please know it is PLOS ONE policy for corresponding authors to declare, on behalf of all authors, all potential competing interests for the purposes of transparency. PLOS defines a competing interest as anything that interferes with, or could reasonably be perceived as interfering with, the full and objective presentation, peer review, editorial decision-making, or publication of research or non-research articles submitted to one of the journals. Competing interests can be financial or non-financial, professional, or personal. Competing interests can arise in relationship to an organization or another person. Please follow this link to our website for more details on competing interests: http://journals.plos.org/plosone/s/competing-interests
Response: We have included the amended Competing Interests Statement above in '3a' in the cover letter.
Reviewers' comments:
Reviewer #1:
1. In Abstract section, method section needs to rewrite scientifically. Does the children was sample or parents of the children.
Response: We have revised the method section in the abstract to clarify the sampling method. The parent study used a two stage sampling by first selecting the schools. Then a random sample of school students were selected in the age category that was needed for the study and subsequently we reached out to the parents of the selected students. We have not provided details of the sampling procedure and the questionnaire in the abstract due to word limits by the journal. The revised methods in the abstract reads as (line 26-32):
" A cross-sectional survey was conducted among a random sample of 1599 parents living in urban and rural areas of Mysore district, India. Applying two-stage probability proportionate-to-size sampling, adolescent girls attending 7th through 10th grades in public, private and religious schools located in the district were selected to take home a questionnaire to their parents. Parents were asked whether their children had received one dose of bacillus Calmette–Guérin; three doses of Diphtheria, Pertussis, Tetanus; three doses of oral polio vaccine; and one dose of measles-containing vaccine. In addition, parents were asked about their attitudes towards childhood vaccination."

2. In the introduction section, from line number 86 to 92 "Therefore, it is imperative to……………………. immunization rates" seems the aim and objective of the study needs to be placed at the end of the introduction section.

Response: We have rephrased the text "Therefore, it is imperative to……………………. immunization rates" and moved it to the last paragraph in the introduction before the text about the objectives of the study (line 120-122).
The revised text reads as "Knowing sociodemographic, environmental and psychosocial correlates of childhood vaccination will inform the choice of interventions to reduce parental hesitancy to childhood vaccination. The objectives of this study were …….for analyzing childhood vaccination data."

3. As the study intended to examine health belief theory controlled and regulated on individual compositional and contextual factors, it is highly appreciated if authors can write it separately under the section "Health Belief Theory and Immunization Practices". Line no 101 to 112. use no vaccination not unvaccinated.

Response: we have added a paragraph summarizing information about use of Health Belief Theory and Immunization practices. It reads as follows (line 107-119):

"HBT has been used to explain the predictors of vaccination uptake related to influenza, HPV, measles, pneumonia, and meningitis in different populations [29-31]. According to the HBT, six factors including perceived susceptibility to disease, perceived severity of a disease, expected benefits of immunization, concerns or cost associated with immunization, strategies or information sources/media that promote immunization and confidence to adopt/accept immunization would likely affect the chance of getting a vaccine [28]. Children will likely be vaccinated if parents perceive that their children are susceptible to severe diseases, believe that the benefit their children receive through immunization (e.g., reduce susceptibility to severe diseases) outweighs the cost/concerns about the vaccine, and children appear open to receiving the vaccination [28]. HBT has however, been mostly used as a framework for explaining vaccination in Western countries. In addition, the theory lacks the socio-cultural factors which could affect individuals' beliefs and behaviors related to immunization. There remains limited information on the utility of the HBT to explain parental attitudes and beliefs about childhood vaccination in developing countries like India."

We have also replaced unvaccinated with 'no vaccination' throughout the manuscript (line 200-206 and line 195 (Table 2).

4. Line no 112 onwards is existing gap and support for your argument to examine HBT framework in Indian scenario followed by objectives. So, the line number 86 to 92 will also be part of this section.

Response: we have rephrased the text from line number 86 to 92 in the older version of the manuscript "Therefore, it is imperative to……………………. immunization rates" and moved it to the last paragraph in the introduction before the text about the objectives of the study. We used the text to support the rationale for the study.

5. Overall, Introduction section needed to rewrite with proper flow setting, as the line no 123-124 should not be part of this section.

Response: We have made major revisions in the introduction following your suggestions above and below. Please see comment 15 below for additional revisions we have made in the introduction. (line 120-122).
6. In the Materials and Method section, Study design needs clarification. As the authors have selected adolescent girl children. In Indian scenario, gender dimension works differently. For the purpose authors are advised to go through gender biasness in immunization in India. As within demographic and health studies in developing counties including India have found disparity, discrimination and exclusion regulated by socio-economic and cultural factors contribute majorly in resource allocation and achievement which lead to poor outcome for female (child, adolescent and adult). Had the authors all these issues in mind when they were finalizing the sampling frame. So within the HBT framework, gender dimension needs to be included. Even it has not discussed in HBT framework section needs attention.
Response: We have revised the study design and placed it under the heading 'Study design and participants recruitment procedure'. It reads as follows:
"A cross-sectional study was conducted among a random sample of parents whose daughters aged 11 to 15 years were attending 7th through 10th grades in schools located in the urban and rural areas of Mysore District. In order to increase the chance of inclusion of parents with a variety of backgrounds while ensuring representativeness of the samples, two-stage probability proportionate-to-size sampling was applied to select adolescent girls attending public, private and religious schools located in the district.
Study participants recruitment procedures are explained in detail elsewhere [33,34]. In brief, 12 schools in the urban area (five government, four private and three religious) and 11 schools in the rural areas (10 government and one private) of Mysore District were selected. A program announcement was sent home with all girls in 7th through 10th grades in the selected schools, explaining the objectives of the study and inviting eligible parents to participate. Of all the girl students in the selected age group in the

select schools, 800 girls from urban schools, and 850 girls from rural schools were randomly selected and provided with a questionnaire and consent form to take home to their parents. The girls were expected to return the completed questionnaire and signed consent form within a week. Only one parent was required to complete the questionnaire. A small proportion of parents in the urban (2.7%) and rural (2.2%) areas did not return the completed questionnaire and/or the sign the consent form. IRB approval for this study was obtained from Florida International University and Public Health Research Institute of India."
We have also discussed the limitations of the generalizability of the current finding due to potential gender bias in immunization in India. It reads as follows (Line 428-431): "Finally, we are reporting on data collected from parents who had adolescent daughters attending school during the study period. As a result, the generalizability of the current results might be limited to parents with at least one school-going adolescent daughter. The generalizability of the study findings to parents without school-going adolescent girls or with only school-going boys will be further limited due to gender biases in immunization in India [52,53]."
7. Further, the question is why the authors had selected female children of age 11-15 years, if they were interested in examining the regulators and predictors of basic immunization in Mysore. As per the guidelines, the basic immunization what the authors have mentioned can be completed within the age of one year as the measles vaccine is given at last after completing the age of 9 months. So, question arises that perception about vaccination may have altered over the period of 10-14 years due to various factors. So, how this HBT can be applied, is another concern.
Response: This was a secondary analysis of data from a study implemented to examine HPV vaccine acceptance of parents in India. The primary goal of the project was to examine factors affecting intention to accept HPV vaccine for the girls as the HPV vaccine is only licensed for them in India. Assessing factors correlated with childhood vaccination was a secondary objective of the project. As the Indian government approved HPV vaccination to only adolescent girls during the study period, we targeted parents who have at least one adolescent daughter during the study period. To reach out to these parents effectively, we selected adolescent girls attending schools during the study period. However, we acknowledge that since the study participant was a parent of atleast one adolescent daughter, there is a potential for selection bias and information bias as we are asking them about events that took place more than ten years ago, and this may limit the applicability of the HBT to explain factors predicting childhood vaccination in the Indian population. We have discussed this as a limitation of the study. It reads as follows (line 428-437): "Finally, we are reporting on data collected from parents who had adolescent daughters attending school during the study period. As a result, the generalizability of the current results might be limited to parents with at least one school-going adolescent daughter. The generalizability of the study findings to parents without school-going adolescent girls or with only school-going boys will be further limited due by gender biases in immunization in India [52,53]. It is also possible that there may be recall bias and parental attitudes may have changed about childhood vaccinations. Thus, policy measures designed to change negative attitudes and beliefs of parents about childhood vaccination based on the current results should be done carefully considering potential changes over the period of time."

8. Further, are all the selected schools government or private? As the affordability to cater and send the children to these two type of school matters more. It is also important to add here that there is high possibility of very few private schools in rural areas. So, the authors have to mention the selection criteria for school is also needed.
Response: In order to include parents with a wide range of background and increase the representativeness of the sample to the population in the study area, we have included parents of adolescents who were attending government, private and religious schools. The schools were selected randomly using proportionate to size. The revised text about the type of schools selected and the selection procedure reads as follows: (line 138-145)
"In order to increase the chance of inclusion of parents with a variety of backgrounds while ensuring representativeness of the samples, two-stage probability proportionate-to-size sampling was applied to select adolescent girls attending public, private and religious schools located in the district. Study participants recruitment procedures are explained in detail elsewhere [33,34]. In brief, 12 schools in the urban area (five government, four private and three religious) and 11 schools in the rural areas (10

government and one private) of Mysore district were selected based on probability proportionate to size sampling."."

9. Authors have mentioned about majority is living in rural areas. As per the 2011 census, close to 69% of country population and 61% of state population was living in rural areas. In case of Mysore, it is close to 58 percent. So using majority for Mysore district is not appropriate here.
Response: We thank the reviewer for this comment and have replaced majority with 58.5%. The revised text reads as: "Of the 3,001,127 people living in the district (density=450/km2), 58.5% live in rural areas (1,755,714)". (line 133, 134)

10. In the text author have mentioned four categories for each of the facilitators and barriers for the child vaccination but in the table option, only three categories is provided. The table can be in appendix section.
Response: We have corrected the response categories text in the table. We have removed the column with response categories in the table and provided that same information as a footnote in the table. It reads as follows: 'Responses to all the items were recorded in four categories: 0= No, 1= Not sure, 2= Yes, 3= Not applicable."
11. In the line number 142 "Only one parent in each family was required to complete the questionnaire". It seems fine but In Indian scenario, how is it possible that 112 are unmarried in table 2 who are having girl child/children of age 11-15 years. Are they male or female?. As per the knowledge and understanding, In India, without marriage, giving birth was not acceptable. However, there may be possibilities that if the parent are unmarried, they would have adopted it or else. So, authors have to clearly mention about who are those single parent.
Response: We thank the reviewer for pointing out this statement. We want to clarify that we have included the definition for 'single parent' in Table 2 as a footnote. It now reads as "Single=unmarried or separated or widowed". Most of the parents who reported their marital status as single were separated or widowed.
12. Line No 191-202 is contextualizing and purpose of use of SEM to model and examine the magnitude and direction of facilitator and barrier predictors controlled on Parent SES characteristics, seems fine but need to rewrite in scientific manner. In the next section, it seems human error has happened as sign of 'greater than' and 'lesser than' is incorrect.
Response : We thank the reviewer for catching this error. We have revised the text about the SEM and have corrected the sign of 'greater than' and 'less than in the data analysis on the method section. It reads as follows (line 227-246):
"SEM was used to examine parental perceptions that directly predicted childhood vaccination, and sociodemographic factors that indirectly predicted the behavior. SEM was also used to evaluate the mediating roles of parental perceptions on the relationship between sociodemographic factors and childhood vaccination and the moderating role of 'rural or urban residence' on the relationship between sociodemographic factors and parental perceptions about childhood vaccination. Moreover, SEM was applied to test model fit of the proposed HBT derived model (Fig 1) with the observed data.
Model fit was checked using chi-square (model acceptable if $P > 0.05$) and other fit indices including Comparative Fit Index (model acceptable if $CFI > 0.90$), Tucker–Lewis Index (model acceptable if $TLI > 0.90$), and Root Mean Square Error of Approximation (model acceptable if RMSEA is < 0.08) [38]. Before fitting the SEM model that has the measurement and structural components in it, fit of the measurement model that included the constructs' perceived benefits/facilitators' and 'barriers' to childhood vaccination was checked. In order to improve the fit of the measurement model, a slight modification was made by removing items with factor loading < 2 and by allowing some of the items to freely co-vary. Similarly, we introduced slight changes in the SEM model based on the Mplus model fit indices output [39]. Since the outcome variable (full vaccination status) was categorical in nature, the Weighted Least Squares Estimation Method was used to estimate the parameters (path coefficients, factor loadings, variances,and covariances) and model fit statistics [40]. Standard errors and 95% CI for direct and indirect effects were estimated using the delta method [41]."
13. Measurement model and structural model can be single section and need methodological and scientific rigor and elaboration.
Response: We have merged the text about the measurement and structural model under a single heading (Structural equation model) and revised the text to better

explain the model. The revised text about the mode reads as follows (line 259-307):
Structural Equation Model
"The measurement model for the construct 'perceived benefits/facilitators to childhood vaccination' that included all the items listed in table 1 fitted acceptably to the data based on the RMSEA. But the model showed lack of fit based on CFI and TLI values (RMSEA= 0.05, CFI= 0.89, TLI= 0.86). After allowing some residual terms for items to covary (F1 WITH F2; F3 WITH F4; F10 WITH F11; F10 WITH F12; F11 WITH F12), fit of the construct measurement model 'perceived benefits/facilitators to childhood vaccination' improved (RMSEA=0.03, CFI=0.97, TLI=0.96). All 12 items listed in Table 1 were used to measure the construct 'perceived benefits/facilitators' that significantly loaded with standardized factor loadings (β) ranging from 0.27 to 0.75. The items "It is very important that my children receive all their vaccine" and "I have a responsibility to have my children vaccinated for the protection of all children" explained sufficient variance or strongly influenced/correlated (β = 0.75 and 0.73, respectively), and the item "Parents should make health decision for their own children rather than a doctor" explained small variance weakly influenced/correlated (β = 0.27) with the score for the construct variable 'perceived benefits/facilitator' to childhood vaccination. The remaining nine items moderately influenced/correlated with the score of the construct variable perceived benefits/facilitator (β = 0.43 to 0.67).
The measurement model for the construct 'perceived barriers to childhood vaccination' fitted acceptably to the data (RMSEA= 0.06, CFI= 0.94, TLI= 0.92, β=0.12 to 0.66), but the factor loading index for the item 'I would feel responsible, if anything bad happened I had my child vaccinated' was 0.12. After removing items with a factor loading less than two, and allowing the residual terms for some items to covary (B5 WITH B6; B6 WITH B8), data fit of the measurement model for the construct 'barriers' to childhood vaccination improved (RMSEA= 0.05, CFI=0.98, TLI=0.96). Seven items, B1- B3 & B5 - B8 used to measure the construct 'perceived barriers' significantly loaded with a standardized factor loading ranging from 0.20 to 0.73. The item "Getting time off from work or household duties makes it difficult to take my child for vaccination" explained sufficient variance or strongly influenced/correlated (β = 0.73) and the item "I am concerned about vaccine side effects" explained small variance or weakly influenced/correlated (β = 0.20) with the score for the construct variable perceived barriers to childhood vaccination. The observed scores for the remaining four items moderately influenced/correlated with the score of the construct variable perceived benefits/facilitator to childhood vaccination (β = 0.43 to 0.67). The item "It is better to get the disease and protected" showed a modest-sized positive influence/correlation (β = 0.35) with the construct variable perceived benefits/facilitator to childhood vaccination. The final measurement model that included 19 items used to measure facilitators and barriers to childhood vaccination, had acceptable fit the data. (RMSEA=0.04, 95% CI: 0.03 - 0.04; CFI=0.93, TLI=0.91) (Fig 2).
Fig 2. Measurement model: factor loadings of items used to measure facilitators and barriers about childhood vaccination in Mysore, India 2010/2011.
The final full SEM model with the structural and measurement components was identified. The total number of free parameters estimated (#130) was less than the number of model parameters (#630). The number of free parameters was the sum of the numbers of estimates, including factor loadings, variances of error and covariance. The number of model parameters is the number of unique covariances of measured variables estimated as the product of the number of measured variables (#35) (the number of measured variables+1) divided by 2.
Fit of the final SEM model to the data was also acceptable based on RMSEA, CFI and TLI values (RMSEA: 0.02: 90% CI: 0.018 to 0.023, CFI=0.92; TLI=0.91) (Fig 3). The chi-square statistic however, suggested a significant difference in the covariance matrix in the proposed model (Fig 1) and the observed data ($\chi2$=3914.6, DF=490, $p<0.01$).
Fig 3. Structural Equation Model showing moderators, mediators and direct and indirect predictors of childhood vaccination in Mysore, India, 2010/2011."

14. It is also observed that when author is using coefficient value, used "=" sign for p values, needs correction at many places.
Response: We have corrected use of the = sign (added space) and 'p' (capitalized, italicized and added space) in the revised manuscript.
15. As the author used SEM to depict facilitator and barriers in child vaccination moderated by parents SES suing primary data collected from Mysore, authors should add why it is more appropriate in the study as other methods like path analysis,

simultaneous equation modelling and CFA etc.
Response: We have revised the text in the introduction to give readers why SEM is a more appropriate method to model predictors of childhood vaccination and explain the relationship between the predictors effectively as compared to other methods including logistic regression analysis, path analysis, simultaneous equation modelling and CFA. The revised text reads as follows (Line 84-107):
"Most of these studies however assumed direct relationships between sociodemographic, environmental, psychological factors and childhood vaccination in logistic regression models [12-26]. On the other hand, some predictors of childhood vaccination may have a direct effect, and some may have an indirect effect. Furthermore, some may play a mediating or moderating role between the predictors and childhood vaccination [27,28].

Evidence based on Health Belief Theory (HBT) suggests that factors that affect preventive behaviors such as vaccination are complex and multifaceted, and do not always act in similar manners [27,28]. According to HBT, individual perceptions about the benefits and barriers to behavior would directly affect the practice of behavior, the effect of sociodemographic status on behavior on the other hand, would be indirect, influencing individual perceptions about the benefits and barriers of behavior [27,28]. In addition, as sociodemographics vary between urban and rural residents in India, [8,9], we hypothesize that the effect of sociodemographic factors may vary by place of residence (urban and rural). Despite the availability of health frameworks that can help us examine factors affecting childhood vaccinations in a comprehensive manner, most studies on childhood vaccination in India did not use a theoretical framework [12-26]. Moreover, some predictors of childhood vaccination can be directly observed, as are some latent variables or constructs [27,28]. As a result, methods including standard logistic regression, factor analysis, simultaneous equation modelling and path analysis may not always be appropriate to model predictors of childhood vaccination, and exploring the relationships between the predictors. Health theory-driven complex models that employ robust analytic techniques (e.g. Structural Equation Model) are needed to better explain the nature of the relationship between sociodemographic, attitudinal and environmental factors while predicting correlates of routine childhood vaccination."

16. In the discussion section, the statement made needs clarification and updation as well. Line no-313 to line no 320 needs correction. As the sriva statava study is based on third round of DLHS survey data of India conducted during 2007-08, not at three consecutive periods of DLSH-1, DLHS-2 and DLHS-3. Further, as the author is submitting the paper in 2020, the latest available data and report for NFHS is for 2015/16 and it also provide estimates at district level for many indicators including for immunization.
Response: We are grateful for the reviewer for finding this typo. We have revised the results of the study by Shrivastwa, et al. Vaccine. 2015;33:D99-105
The revised text in the discussion reads as follows (line 375-377): "A pooled analysis of 108,057 Indian children aged 12–23 months during a national survey for the period 2007–08 reported similar rates of full childhood vaccination of 65.6% for urban vs 53.6% for rural, residents."
We have also discussed and compared the current finding on full vaccination rate between urban vs rural residents with the Indian National Family Health Survey data collected during 2015–2016 and cited the reference. The revised text in the discussion reads as (line 377-380): "Analysis of the National Family Health Survey data collected during 1992–93, 1998–99, 2005–06 and 2015–2016 also showed a lower vaccination rate among rural Indian children compared to those living in urban areas [9, 42, 43]."
Reference: Ministry of Health and Family Welfare. Indian fact sheet: National Family Health Survey (NFHS-4) Accessed from http://rchiips.org/NFHS/pdf/NFHS4/India.pdf in July 6 2020.

17. There is an impression that study examines the HBT in child immunization behaviour using the data from Mysore district, Karnataka and modelled through SEM, but in the result as well as discussion part, detailed on facilitator and barriers seems missing. Although, Parents SES are discussed with more for education.
Response: We have provided details on facilitators and barriers to childhood vaccination in the results and discussion based on the data in table 3 and fig 3. The revised text in the results reads as follows (line 310-328):

	"Full childhood vaccination significantly increased with parental perceptions about the benefits/facilitators to childhood vaccination (standardized regression coefficient (β)=0.29,P <0.001). In other words, it increased if parents felt the vaccine was effective in preventing disease and ensuring child health, feeling a responsibility to protect their child and others, having a healthcare provider recommendation, receiving information about childhood vaccine from a doctor or nurse, knowing where to go for vaccinations, perceiving that the government does a good job providing vaccines and health services, and having several vaccines included in the childhood vaccination schedule. Parental education was significantly associated indirectly with increased full vaccination (β=0.08, P<0.001). Parental employment was indirectly associated with a decreased rate of full vaccination (β= -0.05, P = 0.05). The relationship of parental education (β=0.08, P<0.001) and employment (β=-0.06, P = 0.045) with the rate of full vaccination were significantly mediated by parental perceptions about the benefits/facilitators to childhood vaccination. Parental perceptions about barriers to childhood vaccination neither showed a significant association directly with the rate of full vaccination nor mediated the relationship between sociodemographic variables including age, gender, marital status, religion, education, occupation and number of children, and the rate of full vaccination (Table 3). In addition, age, gender, marital status, religion and number of children in a family had no indirect relationship with the rate of full vaccination through the construct about perceptions about the benefits/facilitators (Table 3)."<br><br>The revised/added text about the influence of perceived benefits/facilitators on childhood vaccination and its implications for intervention in the discussion reads as follows (line 384 to 412):<br>"Parents perceiving that childhood vaccination was beneficial (e.g. effective in preventing disease, ensure their child health, help protect other children); those who felt responsible if anything bad happened did not have their child vaccinated, and those who heard about the vaccine or received a positive recommendation from a doctor or nurse, were more likely to report full vaccination. In addition, parental attitudes about childhood vaccination played a mediating role between sociodemographic characteristics and 'full vaccination' rate and this conquered with a previous report [44]. A number of studies in India, Pakistan, Nigeria, Benin and Uganda reported similar results for the relationship between levels of vaccination and parental attitudes and beliefs related to immunization [45,46]. Similarly, a UK study showed that the need to protect children and help protect others through herd immunity, were seen to positively influence parental decisions about vaccinations [47].<br><br>These findings indicate key issues in the perceived benefits domain of HBT, that could be leveraged to improve childhood vaccination rates. Community-based education campaigns focused on increasing parental awareness about the benefits of childhood vaccination in preventing disease and promoting child health may be useful. In addition, strategies led by physicians and nurses should focus on strong childhood vaccination recommendations. The psychological decision-making frameworks suggested that when individuals are uncertain, they are more open to information about vaccination in their decision-making [48]. This study also suggests that intervention to increase vaccination in children may target change in parental perception about the benefits of vaccination in protecting others, which agree with the vaccine decision-making framework [47]."<br><br>Overall, authors are advised to restructure the paper as per framework mentioned and modeled using SEM. Sampling design written should be valid and justified. Methodology section needs linking with framework and the limitations of the same. Result and discussion section should more focus on facilitator and barriers how regulated by HBT is another concern of the paper. Authors should take care of grammatical errors and editing of the manuscript.<br>Response: We have followed your suggestions above and made the necessary changes in the abstract, introduction, methods, results and discussion in this revised version of the manuscripts. Hopefully, these changes will address your concerns.<br><br>We have also further edited the manuscript for grammar and language.
**Additional Information:**	

Question	Response
**Financial Disclosure**<br><br>Enter a financial disclosure statement that describes the sources of funding for the work included in this submission. Review the submission guidelines for detailed requirements. View published research articles from *PLOS ONE* for specific examples.<br><br>This statement is required for submission and **will appear in the published article** if the submission is accepted. Please make sure it is accurate.<br><br>**Unfunded studies**<br>Enter: *The author(s) received no specific funding for this work.*<br><br>**Funded studies**<br>Enter a statement with the following details:<br>• Initials of the authors who received each award<br>• Grant numbers awarded to each author<br>• The full name of each funder<br>• URL of each funder website<br>• Did the sponsors or funders play any role in the study design, data collection and analysis, decision to publish, or preparation of the manuscript?<br>• **NO** - Include this sentence at the end of your statement: *The funders had no role in study design, data collection and analysis, decision to publish, or preparation of the manuscript.*<br>• **YES** - Specify the role(s) played.<br><br>* typeset	This study was funded by an Investigator-Initiated Award from Merck & Co., Inc. awarded to PM. KK was funded as a US Scholar by Fogarty International Center and National Heart Lung and Blood Institute, and National Institute of Neurological Disorders and Stroke and of the National Institutes of Health under Award Number D43 TW010540. PM was partially funded by grants from National Institutes for Health (D43-TW010540; R15-AI128714). The funder had no role in the study design, data collection, analysis, interpretation or publication of the manuscript."
**Competing Interests**<br><br>Use the instructions below to enter a competing interest statement for this submission. On behalf of all authors, disclose any competing interests that could be perceived to bias this work—acknowledging all financial support and any other relevant financial or non-financial competing interests.<br><br>This statement **will appear in the published article** if the submission is	This study was awarded from Merck & Co., Inc. This does not alter our adherence to PLOS ONE policies on sharing data and materials

accepted. Please make sure it is accurate. View published research articles from *PLOS ONE* for specific examples.<br><br>**NO authors have competing interests**<br><br>Enter: *The authors have declared that no competing interests exist.*<br><br>**Authors with competing interests**<br><br>Enter competing interest details beginning with this statement:<br><br>*I have read the journal's policy and the authors of this manuscript have the following competing interests: [insert competing interests here]*<br><br>* typeset	
**Ethics Statement**<br><br>Enter an ethics statement for this submission. This statement is required if the study involved:<br><br>• Human participants<br>• Human specimens or tissue<br>• Vertebrate animals or cephalopods<br>• Vertebrate embryos or tissues<br>• Field research<br><br>Write "N/A" if the submission does not require an ethics statement.<br><br>General guidance is provided below. Consult the submission guidelines for detailed instructions. **Make sure that all information entered here is included in the Methods section of the manuscript.**	IRB approval for this study was obtained from Florida International University and Public Health Research Institute of India, and parents provided written informed consent.

**Format for specific study types**<br><br>**Human Subject Research (involving human participants and/or tissue)**<br>• Give the name of the institutional review board or ethics committee that approved the study<br>• Include the approval number and/or a statement indicating approval of this research<br>• Indicate the form of consent obtained (written/oral) or the reason that consent was not obtained (e.g. the data were analyzed anonymously)<br><br>**Animal Research (involving vertebrate animals, embryos or tissues)**<br>• Provide the name of the Institutional Animal Care and Use Committee (IACUC) or other relevant ethics board that reviewed the study protocol, and indicate whether they approved this research or granted a formal waiver of ethical approval<br>• Include an approval number if one was obtained<br>• If the study involved *non-human primates*, add *additional details* about animal welfare and steps taken to ameliorate suffering<br>• If anesthesia, euthanasia, or any kind of animal sacrifice is part of the study, include briefly which substances and/or methods were applied<br><br>**Field Research**<br><br>Include the following details if this study involves the collection of plant, animal, or other materials from a natural setting:<br>• Field permit number<br>• Name of the institution or relevant body that granted permission	
**Data Availability**<br><br>Authors are required to make all data underlying the findings described fully available, without restriction, and from the time of publication. PLOS allows rare exceptions to address legal and ethical concerns. See the PLOS Data Policy and FAQ for detailed information.	Yes - all data are fully available without restriction

Powered by Editorial Manager® and ProduXion Manager® from Aries Systems Corporation

A Data Availability Statement describing where the data can be found is required at submission. Your answers to this question constitute the Data Availability Statement and **will be published in the article**, if accepted.<br><br>**Important:** Stating 'data available on request from the author' is not sufficient. If your data are only available upon request, select 'No' for the first question and explain your exceptional situation in the text box.<br><br>Do the authors confirm that all data underlying the findings described in their manuscript are fully available without restriction?	
**Describe where the data may be found in full sentences. If you are copying our sample text, replace any instances of XXX with the appropriate details.**<br><br>• If the data are **held or will be held in a public repository**, include URLs, accession numbers or DOIs. If this information will only be available after acceptance, indicate this by ticking the box below. For example: *All XXX files are available from the XXX database (accession number(s) XXX, XXX.*).<br>• If the data are all contained **within the manuscript and/or Supporting Information files**, enter the following: *All relevant data are within the manuscript and its Supporting Information files.*<br>• If neither of these applies but you are able to provide **details of access elsewhere**, with or without limitations, please do so. For example:<br><br>*Data cannot be shared publicly because of [XXX]. Data are available from the XXX Institutional Data Access / Ethics Committee (contact via XXX) for researchers who meet the criteria for access to confidential data.*<br><br>*The data underlying the results presented in the study are available from (include the name of the third party*	All relevant data are within the manuscript.

and contact information or URL).<br>• This text is appropriate if the data are owned by a third party and authors do not have permission to share the data.<br><br>* typeset	
Additional data availability information:	

Powered by Editorial Manager® and ProduXion Manager® from Aries Systems Corporation

Manuscript
Click here to access/download;Manuscript;Manuscript_clean copy.docx

Structural equation modeling to detect correlates of childhood vaccination: a moderated mediation analysis

Abraham Degarege[1]; Karl Krupp[2,3]; Vijaya Srinivas[3]; Boubakari Ibrahimou[4]; Purnima Madhivanan[2,3,5,6]

[1] Department of Epidemiology, College of Public Health, University of Nebraska Medical Center, Omaha, Nebraska, USA.

[2] Department of Health Promotion Sciences, Mel & Enid Zuckerman College of Public Health, The University of Arizona, Tucson, Arizona, USA

[3] Public Health Research Institute of India, Mysore, Karnataka, India

[4] Department of Biostatistics, Robert Stempel College of Public Health, Florida International University, Miami, Florida, USA.

[5] Division of Infectious Diseases, College of Medicine, The University of Arizona, Tucson, Arizona, USA

[6] Department of Family & Community Medicine, College of Medicine, The University of Arizona, Tucson, Arizona, USA

Corresponding Author

Abraham Degarege, MS, PhD

Assistant Professor

Department of Epidemiology, University of Nebraska Medical Center

Email: abraham.mengist@unmc.edu Phone: (1) 402-552-6682

Abstract

Objectives

This study used a Health Belief Theory derived framework and Structural Equation Model to examine moderators, mediators and direct and indirect predictors of childhood vaccination.

Methods

A cross-sectional survey was conducted among a random sample of 1599 parents living in urban and rural areas of Mysore district, India. Applying two-stage probability proportionate-to-size sampling, adolescent girls attending 7th through 10th grades in public, private and religious schools located in the district were selected to take home a questionnaire to their parents. Parents were asked whether their children had received one dose of bacillus Calmette–Guérin; three doses of Diphtheria, Pertussis, Tetanus; three doses of oral polio vaccine; and one dose of measles-containing vaccine. In addition, parents were asked about their attitudes towards childhood vaccination.

Results

Out of the 1599 parents, 52.2% reported that their children had received all the routine vaccines (fully vaccinated); 42.7% reported their children had missed at least one routine vaccine, and 5.2% reported that their children had missed all routine vaccinations. Perceptions about the benefits/facilitators to childhood vaccination significantly predicted the full vaccination rate (standardized regression coefficient (β) =0.29) directly and mediated the effect of parental education (β =0.11) and employment (β = -0.06) on the rate of full vaccination. Parental education was significantly associated indirectly with higher rates of full vaccination (β =0.11).

Parental employment was significantly associated indirectly with decreasing rates of full vaccination (β = -0.05). Area of residence moderated the role of religion (β = 0.24) and the 'number of children' in a family (β =0.33) on parental perceptions about barriers to childhood vaccination. The model to data fit was acceptable (Root Mean Square Error of Approximation = 0.02, 95% CI 0.018 to 0.023; Comparative Fit Index = 0.92; Tucker–Lewis Index = 0.91).

Conclusions

Full vaccination rate was relatively low among children in Mysore, especially among parents who were unsure about the benefits of routine vaccination and those with low educational levels. Interventions increasing awareness of the benefits of childhood vaccination that target rural parents with lower levels of education may help increase the rate of full childhood vaccination in India.

Keywords: Childhood vaccination, Structural equation model, Attitude, Parents, India

Introduction

Childhood immunization remains one of the most cost-effective and widely used strategies to reduce morbidity and mortality from vaccine-preventable diseases. Annually, vaccination prevents the death of two to three million children worldwide [1]. More child deaths can be prevented by further improving vaccination levels, coverage, and timeliness [1]. In order to reduce childhood mortality, the World Health Organization (WHO) launched the Expanded Program on Immunization (EPI) in 1974 with the goal of increasing immunization worldwide [2]. In 2012, the WHO proposed ambitious goals to immunize 90% of infants with three doses of diphtheria-tetanus-pertussis (DTP3) vaccine by the year 2020 [3]. The goal remains aspirational in many developing countries [1]. As of 2018, roughly 19.8 million children did not receive routine childhood vaccines [1].

India has the world's largest number of annual births and the lowest childhood immunization rate [4]. While India has made large strides in immunization during the last three decades, immunization levels remain below the WHO goal of ≥90% coverage for DTP3 vaccines by 2020 [3]. India's Universal Vaccination Program (UIP) provides one dose of Bacillus Calmette–Guérin (BCG), three doses of DPT3, three doses of oral polio vaccine (OPV), and one dose of measles-containing vaccine (MCV) to all infants younger than one year for free [5]. Indian children receiving all four routine vaccines at recommended doses are considered fully vaccinated by WHO. Children receiving fewer than recommended doses or missing any of the childhood vaccines are categorized as 'under-vaccinated/partially vaccinated', and those not receiving any vaccinations are considered 'non-vaccinated'.

Incomplete or delayed vaccination against childhood diseases can lead to increased mortality and morbidity in children [6]. Reducing child deaths through immunization requires that adequate numbers of children receive full vaccination in a timely manner [6]. As of 2016, only about 62.0% of children aged 12-23 months were fully vaccinated in India [7]. India has a large and heterogeneous population with varied cultures, sociodemographics, religion, and education status, leading to variations in vaccination levels among regions [8,9,10,11]. The presence of under-vaccinated groups in some regions may be a source of infectious disease outbreaks in the country.

Previous studies in India have examined demand/individual-related factors affecting routine vaccination [12-26]. Most of these studies, however, assumed direct relationships between sociodemographic, environmental, psychological factors and childhood vaccination in logistic regression models [12-26]. On the other hand, some predictors of childhood vaccination may have a direct effect, and some may have an indirect effect. Furthermore, some may play a mediating or moderating role between the predictors and childhood vaccination [27,28].

[illegible]vidence-based on Health Belief Theory (HBT) suggests that factors that affect preventive behaviors such as vaccination are complex and multifaceted, and do not always act in similar manners [27,28]. According to HBT, individual perceptions about the benefits and barriers to behavior would directly affect the practice of behavior; the effect of sociodemographic status on behavior, on the other hand, would be indirect, influencing individual perceptions about the benefits and barriers of behavior [27,28].

In addition, as sociodemographics vary between urban and rural residents in India, [8,9], we hypothesize that the effect of sociodemographic factors may vary by place of residence (urban and rural). Despite the availability of health frameworks that can help us examine factors

Summary of Comments on PONE-D-20-09473_R1_reviewer.pdf

Page: 19

Number: 1

This hyphen should be removed.

affecting childhood vaccinations in a comprehensive manner, most studies on childhood vaccination in India did not use a theoretical framework [12-26]. Moreover, some predictors of childhood vaccination can be directly observed, as are some latent variables or constructs [27,28]. As a result, methods including standard logistic regression, factor analysis, simultaneous equation modeling and path analysis may not always be appropriate to model predictors of childhood vaccination and exploring the relationships between the predictors. Health theory-driven complex models that employ a robust analytic technique (e.g. Structural Equation Model) are needed to better explain the nature of the relationship between sociodemographic, attitudinal and environmental factors while predicting correlates of routine childhood vaccination.

HBT has been used to explain the predictors of vaccination uptake related to influenza and HPV in different populations [29-31]. According to ~~the~~ HBT, six factors including perceived susceptibility to disease, perceived severity of a disease, expected benefits of immunization, concerns or cost associated with immunization, strategies or information sources/media that promote immunization and confidence to adopt/accept immunization would likely affect the chance of getting a vaccine [28]. Children will likely be vaccinated if parents perceive that their children are susceptible to severe diseases, believe that the benefit their children receive through immunization (e.g., reduce susceptibility to severe diseases) outweighs the cost/concerns about the vaccine, and children appear open to receiving the vaccination [28]. HBT has, however, been mostly used as a framework for explaining vaccination in Western countries. In addition, the theory lacks the socio-cultural factors which could affect individuals' beliefs and behaviors related to immunization. There remains limited information on the utility of the HBT to explain parental attitudes and beliefs about childhood vaccination in developing countries like India.

Page: 20

Number: 1

No need to capitalize

Number: 2

Number: 3

Would be interesting to add at least a sentence describing why HBT has been applied disproportionately to Western countries.

Knowing sociodemographic, environmental and psychosocial correlates of childhood vaccination will inform the choice of interventions to reduce parental hesitancy to childhood vaccination. The objectives of this study were to i) determine the direct and indirect predictors of childhood vaccination, ii) examine the mediating role of parental attitudes about childhood vaccination on the relationship between sociodemographic factors and childhood vaccination, iii) test the moderating role of the area of residence on the relationship between sociodemographic factors and parental attitudes about childhood vaccination; and iv) test the overall fit of an HBT derived model for analyzing childhood vaccination data.

Materials and Methods

Study area

The study was conducted among a random sample of parents of adolescent [1] girls living [2] in urban and rural areas of Mysore district, India, between February 2010 and October 2011. Mysore district is in the southern part of Karnataka, the 6th largest state by area and the 8th by population size in India. Of the 3,001,127 people living in the district (density = 450/km^2), 58.5% live in rural areas (1,755,714) [32].

Study design and participants recruitment procedure

A cross-sectional study was conducted among a random sample of parents whose daughters aged 11 to 15 years were attending 7th through 10th grades in schools located in the urban and rural areas of Mysore District. In order to increase the chance of inclusion of parents with a variety of backgrounds while ensuring the representativeness of the samples, two-stage probability

Page: 21

Number: 1

Describe why girls specifically

Number: 2 Date: 8/14/2020 11:35:35 AM

residing

proportionate-to-size sampling was applied to select adolescent girls attending public, private and religious schools located in the district.

Study participants recruitment procedures are explained in detail elsewhere [33,34]. In brief, 12 schools in the urban area (five government, four private and three religious) and 11 schools in the rural areas (10 government and one private) of Mysore district were selected based on probability proportionate to size sampling. A program announcement was sent home with all girls in 7th through 10th grades in the selected schools, explaining the objectives of the study and inviting eligible parents to participate. Of all the girl students in the selected age group in the select schools, 800 girls from urban schools, and 850 girls from rural schools were randomly selected and provided with a questionnaire and consent form to take home to their parents. The girls were expected to return the completed questionnaire and signed a consent form within a week. Only one parent was required to complete the questionnaire. A small proportion of parents in the urban (2.7%) and rural (2.2%) areas did not return the completed questionnaire and/or signed the consent form. IRB approval for this study was obtained from Florida International University and Public Health Research Institute of India.

Questionnaire

The questionnaire was administered in *Kannada,* the local language, to assess factors affecting general vaccination and HPV vaccination in particular. A detailed description of the questionnaire is described elsewhere [33-35]. It included items such as "*Please indicate (to the best of your knowledge whether all your children have had the following recommended vaccinations*" to assess immunization for BCG, DPT3, OPV and MCV. Responses were recorded as four categories (0= No, 1= Not sure, 2= Yes, 3= Not applicable). Twenty items

(Table 1) assessed parental attitudes about childhood vaccination, and seven items described the sociodemographic status of parents (gender, age in years, marital status, religion, occupation, educational status, number of children in a family) (Table 2). The 20 items used to examine parental attitudes about childhood vaccination were grouped into two constructs: facilitators/benefits of childhood vaccination (12 items), and barriers to childhood vaccination (8 items). Responses to items assessing parental attitudes about childhood vaccination were recorded in four categories (0= No, 1= Not sure, 2= Yes, 3= Not applicable).

Page: 23

Number: 1

Was there a question about the immunization status of the parents as children?

Table 1. Latent variables/constructs and the corresponding measuring items along with their responses

Constructs		Item
Facilitators to childhood vaccination (F)		
	F1	Vaccination are effective in preventing disease
	F2	It is very important that my children receive all their vaccine
	F3	Vaccine is one way that parents can ensure their child health
	F4	I have a responsibility to have my children vaccinated for the protection of all children
	F5	Parents should make health decision for their own children rather than a doctor
	F6	The government does a good job providing vaccine and health services
	F7	There are many vaccines included in the childhood vaccination schedule
	F8	I would feel responsible if anything bad happened I did not have my child vaccinated
	F9	I would know where to go if I wanted to have my child vaccinated
	F10	When we visit a doctor/nurse, they tell us about vaccinations for my child
	F11	When we visit a doctor/nurse, we ask about vaccination for our child
	F12	We always get the vaccinations recommended by a doctor or nurse
Barriers to vaccination (B)		
	B1	I am concerned about vaccine side effects
	B2	I am afraid of vaccination of my children
	B3	It is better to get the disease and protected
	B4	I would feel responsible if anything bad happened I had my child vaccinated
	B5	The high cost of transportation would affect my decision about whether to vaccinate my child
	B6	Cost is an important factor in deciding whether to vaccinate my child
	B7	Getting time off from work or household duties makes it difficult to take my child for vaccination
	B8	I would not give optional vaccines to my child because it is too expensive

Table 2. Sociodemographic characters of the study participants/parents and vaccination status of their children at a family level in Mysore, India 2010/2011

Sociodemographic Characters of the parents	Categories (n)	Vaccination status of children at a family level			p-value
		Fully vaccinated (%)	Under vaccinated (%)	No vaccination (%)	
Education	No formal education (636)	39.3	53.1	7.55	<0.001
	Grade 1 to 10th (676)	54.0	41.86	4.1	
	High school or above (287)	76.3	21.3	2.4	
Occupation	Unemployed (724)	54.7	40.19	5.11	0.172
	Employed (875)	50.1	44.7	5.3	
Area	Rural (822)	42.6	51.3	6.1	<0.001
	Urban (777)	62.3	33.5	4.3	
Age (years)	≤35 (679)	47.3	46.5	6.2	0.003
	>35 (920)	55.8	39.8	4.5	
Gender	Males (433)	53.6	42.7	3.7	0.248
	Females (1166)	51.6	42.6	5.8	
Religion	Hindus (1420)	51.6	43.2	5.1	0.133
	Muslims (154)	5.1	40.9	4.6	
	Christians (25)	68.0	20.0	12.0	
Marital status	Married (1487)	52.6	42.2	5.2	0.441
	Single (112)*	46.4	48.2	5.4	
Number of children in a family	One (131)	55.0	39.7	5.3	0.773
	Two (719)	55.8	39.6	4.6	
	Three (749)	48.2	46.1	5.7	

Single=unmarried or separated or widowed

Data analysis

The M plus (version 7.3) software package was used for data analysis [36]. The main outcome variable was family childhood vaccination status grouped into three categories: fully vaccinated, under-vaccinated and no vaccination. 'Full vaccination' described families with children that had received one dose of BCG, three doses of DPT, three doses of OPV and one dose of MCV. If at least one child in a family had not received one or more of these vaccines, it was grouped as 'incomplete/under-vaccinated,' and if children had not received any vaccination, the family was grouped as 'no vaccination.'

A modified conceptual model informed by HBT was used to guide the analysis (Fig 1). Rates of full vaccination were predicted by parental perceptions about the benefits/facilitators and barriers to childhood vaccination (Table 1). Parental perceptions about the benefits/facilitators and barriers to childhood vaccination were in turn predicted by sociodemographic variables which included gender (females/males), age group (<35/≥35 years), marital status (unmarried/married), religion (Muslims/Hindus/Christians), occupation (unemployed/employed), educational status (no formal education/formal education), and the number of children in a family (one/two or three). As a number of participants reported 'not applicable' to some of the items used to assess parental attitudes about childhood vaccination were small (<20) or none, they were treated as missing during the analysis. Similarly, the number of participants who reported 'not applicable' or "not sure" were also small (<10), so they were excluded from the analysis.

Fig 1. Proposed Health Belief Theory derived model for examining factors predicting full childhood vaccination.

Page: 26

Number: 1

Would briefly restate that these categories were defined consistently with the WHO criteria

Number: 2

Please give rationale for choosing the age cutoff at 35 years

Number: 3

This seems to be a rushed but potentially important point. The authors imply that the size of this group was small and so would be the impact of their responses on the analysis. This is an overreach (even if it is intuitive) because the impact of their responses was not evaluated.

A confirmatory factor analysis was used to check the reliability and validity of the items used for measuring the constructs of perceived benefits/facilitators and perceived barriers to childhood vaccination. The strength of the influence or correlation of the scores of the items with the scores of the constructs, (i.e. the variability in the scores of the constructs explained by the items) was determined based on the magnitude of the factor loadings for each item [37]. The cut-offs used were ≤ 0.32 (poor), 0.33 - 0.45 (fair), 0.46 - 0.55 (good), 0.56 - 0.69 (very good), ≥ 0.70 (excellent). SEM was used to examine parental perceptions that directly predicted childhood vaccination and sociodemographic factors that indirectly predicted the behavior. SEM was also used to evaluate the mediating roles of parental perceptions on the relationship between sociodemographic factors and childhood vaccination and the moderating role of 'rural or urban residence' on the relationship between sociodemographic factors and parental perceptions about childhood vaccination. Moreover, SEM was applied to test model fit of the proposed HBT derived model (Fig 1) with the observed data.

Model fit was checked using chi-square (model acceptable if $P > 0.05$) and other fit indices including Comparative Fit Index (model acceptable if CFI > 0.90), Tucker–Lewis Index (model acceptable if TLI > 0.90), and Root Mean Square Error of Approximation (model acceptable if RMSEA is < 0.08) [38]. Before fitting the SEM model that has the measurement and structural components in it, fit of the measurement model that included the constructs 'perceived benefits/facilitators' and 'barriers' to childhood vaccination was checked. In order to improve the fit of the measurement model, a slight modification was made by removing items with factor loading < 2 and by allowing some of the items to freely covary. Similarly, we introduced slight changes in the SEM model based on the Mplus model fit indices outputs [39]. Since the outcome variable (full vaccination status) was categorical, the Weighted Least Squares

Estimation Method was used to estimate the parameters (path coefficients, factor loadings, variances, and covariances) and model fit statistics [40]. Standard errors and 95% CI for direct and indirect effects were estimated using the delta method [41].

Results

Characteristics of the study participants

Out of 1650 parents contacted, 19 from rural and 22 from urban areas did not return the questionnaire and/or the signed the consent form. In addition, another ten parents from urban and rural areas responded to the question of uptake of routine childhood vaccines as 'not applicable' or 'not sure'. Data for the remained 1599 parents were analyzed for this study. The majority of study participants were mothers (76.7%), literate (63.4%), reported their religion as Hindus (99.0%), and were aged ≤35 years (53.3%). Rates of full vaccination, under vaccination and non-vaccination among children, were 52.2%, 42.7% and 5.2%, respectively. The rate of full vaccination was significantly greater among parents who were educated (60.6%), urban residents, (62.3%) and those aged >35 years (55.8%) (Table 2).

Structural equation model

The measurement model for the construct 'perceived benefits/facilitators to childhood vaccination" that included all the items listed in table 1 fitted acceptably to the data based on the RMSEA. But the model showed a lack of fit based on CFI and TLI values (RMSEA = 0.05, CFI = 0.89, TLI = 0.86). After allowing some residual terms for items to covary (F1 WITH F2; F3 WITH F4; F10 WITH F11; F10 WITH F12; F11 WITH F12), fit of the construct measurement model 'perceived benefits/facilitators to childhood vaccination' improved

Number: 1

Perhaps I'm missing something but how well can one reply to the questionnaire if she/he is illiterate? Further how might literacy have affected response rates or responses? Did the issue of literacy impact the design or interpretion of this study?

(RMSEA =0.03, CFI =0.97, TLI =0.96). All 12 items listed in Table 1 were used to measure the construct ‘perceived benefits/facilitators’ that significantly loaded with standardized factor loadings (β) ranging from 0.27 to 0.75. The items “*It is very important that my children receive all their vaccine*” and “*I have a responsibility to have my children vaccinated for the protection of all children*” explained sufficient variance or strongly influenced/correlated (β = 0.75 and 0.73, respectively), and the item “*Parents should make health decision for their own children rather than a doctor*” explained small variance weakly influenced/correlated (β = 0.27) with the score for the construct variable ‘perceived benefits/facilitator’ to childhood vaccination. The remaining nine items moderately influenced/correlated with the score of the construct variable ‘perceived benefits/facilitator’ (β = 0.43 to 0.67).

The measurement model for the construct ‘perceived barriers to childhood vaccination’ fitted acceptably to the data (RMSEA = 0.06, CFI = 0.94, TLI = 0.92, β = 0.12 to 0.66), but the factor loading index for the item ‘*I would feel responsible, if anything bad happened I had my child vaccinated*’ was 0.12. After removing items with a factor loading less than two, and allowing the residual terms for some items to covary (B5 WITH B6; B6 WITH B8), data fit of the measurement model for the construct ‘barriers to childhood vaccination’ improved (RMSEA = 0.05, CFI =0.98, TLI =0.96). Seven items, B1- B3 & B5 - B8 used to measure the construct perceived barriers significantly loaded with a standardized factor loading ranging from 0.20 to 0.73. The item “*Getting time off from work or household duties makes it difficult to take my child for vaccination*” explained sufficient variance or strongly influenced/correlated (β = 0.73) and the item “*I am concerned about vaccine side effects*” explained small variance or weakly influenced/correlated (β = 0.20) with the score for the construct variable ‘perceived barriers to childhood vaccination.’ The observed scores for the remaining four items moderately

influenced/correlated with the score of the construct variable ‘perceived benefits/facilitator to childhood vaccination’ (β = 0.43 to 0.67). The item “*It is better to get the disease and protected*” showed a modest-sized positive influence/correlation (β = 0.35) with the construct variable ‘perceived benefits/facilitator to childhood vaccination.’ The final measurement model that included 19 items used to measure facilitators and barriers to childhood vaccination had acceptable fit the data. (RMSEA =0.04, 95% CI: 0.03 - 0.04; CFI =0.93, TLI =0.91) (Fig 2).

Fig 2. Measurement model: factor loadings of items used to measure facilitators and barriers about childhood vaccination in Mysore, India 2010/2011.

The final full SEM model with the structural and measurement components was identified. The total number of free parameters estimated (n=130) was less than the number of model parameters (n=630). The number of free parameters was the sum of the numbers of estimates, including factor loadings, variances of error and covariance. The number of model parameters is the number of unique covariances of measured variables estimated as the product of the number of measured variables (n=35) (the number of measured variables +1) divided by 2.

Fit of the final SEM model to the data was also acceptable based on RMSEA, CFI and TLI values (RMSEA: 0.02: 95% CI: 0.018 to 0.023, CFI=0.92; TLI=0.91) (Fig 3). The chi-square statistic, however, suggested a significant difference in the covariance matrix in the proposed model (Fig 1) and the observed data ($\chi2$ =3914.6, DF=490, $p < 0.01$).

Fig 3. Structural Equation Model showing moderators, mediators and direct and indirect predictors of childhood vaccination in Mysore, India, 2010/2011.

Factors affecting childhood vaccination status

Full childhood vaccination significantly increased with parental perceptions about the benefits/facilitators to childhood vaccination (standardized regression coefficient (β) = 0.29, $P < 0.001$). In other words, it increased if parents felt the vaccine was effective in preventing disease and ensuring child health, feeling a responsibility to protect their child and others, having a healthcare provider recommendation, receiving information about childhood vaccine from a doctor or nurse, knowing where to go for vaccinations, perceiving that the government does a good job providing vaccines and health services, and having several vaccines included in the childhood vaccination schedule. Parental education was significantly associated indirectly with increased full vaccination ($\beta = 0.08$, $P < 0.001$). Parental employment was indirectly associated with a decreased rate of full vaccination ($\beta = - 0.05$, $P = 0.05$). The relationship of parental education ($\beta = 0.08$, $P < 0.001$) and employment ($\beta = -0.06$, $P = 0.045$) with the rate of full vaccination were significantly mediated by parental perceptions about the benefits/facilitators to childhood vaccination. Parental perceptions about barriers to childhood vaccination neither showed a significant association directly with the rate of full vaccination nor mediated the relationship between sociodemographic variables including age, gender, marital status, religion, education, occupation and number of children, and the rate of full vaccination (Table 3). In addition, age, gender, marital status, religion and number of children in a family had no indirect relationship with the rate of full vaccination through the construct about perceptions about the benefits/facilitators (Table 3).

Table 3. Unstandardized (B) and standardized (β) effects of factors affecting full childhood vaccination for the structural model

Factors			Full childhood vaccination	
Direct			*B* (95% CI)	*β* (95% CI)
	Facilitators		**0.62 (0.46, 0.78)**	**0.29 (0.22, 0.36)**
	Barriers		-0.12 (-0.56, 0.33)	-0.02 (-0.10, 0.06)
Indirect		Mediators		
	Age	Facilitators	0.04 (-0.02, 0.09)	0.04 (-0.02, 0.09)
		Barriers	0.002 (-0.007, 0.012)	0.02 (-0.01, 0.01)
		Sum of indirect effect	0.04 (-0.02, 0.10)	0.04 (-0.02, 0.10)
	Gender	Facilitators	0.05 (-0.02, 0.13)	0.05 (-0.02, 0.13)
		Barriers	0.01 (-0.02, 0.03)	0.05 (-0.02, 0.03)
		Sum of indirect effect	0.06 (-0.02, 0.14)	0.12 (-0.02, 0.14)
	Education	Facilitators	**0.11 (0.05, 0.17)**	**0.08 (0.04, 0.12)**
		Barriers	0.01 (-0.01, 0.012)	0.002 (-0.01, 0.01)
		Sum of indirect effect	**0.11 (0.05, 0.17)**	**0.08 (0.04, 0.12)**
	Occupation	Facilitators	**-0.06 (-0.12, -0.001)**	**-0.06 (-0.12, -0.001)**
		Barriers	-0.01 (-0.02, 0.01)	-0.01 (-0.02, 0.012)
		Sum of indirect effect	**-0.06 (-0.11, 0.000)**	**-0.05 (-0.11, 0.00)**
	Religion	Facilitators	-0.07 (-1.33, 1.20)	-0.06 (-1.32, 1.19)
		Barriers	0.01 (-0.02, 0.04)	0.01 (-0.02, 0.04)
		Sum of indirect effect	-0.06 (-1.32, 1.21)	-0.06 (-1.31, 1.20)
	Marital	Facilitators	0.08 (-0.01, 0.17)	0.078 (-0.014, 0.17)
		Barriers	-0.001 (-0.01, 0.01)	-0.001(-0.01, 0.01)
		Sum of indirect effect	0.07 (-0.02, 0.17)	0.07 (-0.015, 0.17)

Effect of background factors on parental perceptions about childhood vaccination

As compared to uneducated parents, those who were educated were more likely to perceive that childhood vaccination was beneficiary ($\beta = 0.37$, $P < 0.001$). Parents living in urban areas were more likely to perceive that childhood vaccination was beneficial than those living in rural areas ($\beta = 0.96$, P=0.022). Employed parents were also less likely to believe that childhood vaccination was beneficial as compared to unemployed ones ($\beta = -0.20$, $P = 0.036$). Perceptions about barriers to childhood vaccination was lower among women than in men ($\beta = -0.27$, $P = 0.030$). When compared to parents with one child, those with two or more children were less likely to perceive that childhood vaccinations were harmful ($\beta = -0.67$, $P = 0.001$). Geographical region significantly moderated the effect of religion (β =0.24, $P = 0.045$), as did number of children (β =0.330, $P = 0.007$) on parental perceptions about barriers to childhood vaccination. However, the nature of the relationship of parental education, gender, age and marital status with parental perceptions about benefits/facilitators and barriers to childhood vaccination did not however vary based on place of residence (Table 4).

Table 4. Standardized effects of sociodemographic factors and interaction terms on parental perceptions about benefits/facilitators and barriers to full childhood vaccination for the SEM

Background (exposure)	Complete childhood vaccination (outcomes)	
	Facilitators<br>*β* (95% CI)	Barriers<br>*β* (95% CI)
Age	0.12 (-0.07, 0.32)	-0.12 (-0.30, 0.07)
Gender	0.18 (-0.07, 0.43)	**-0.27 (-0.52, -0.03)**
Education	**0.37 (0.19, 0.55)**	-0.12 (-0.29, 0.05)
Occupation	**-0.20 (-0.39, -0.013)**	-0.05 (-0.23, 0.13)
Religion	-0.22 (-4.51, 4.07)	-0.37(-1.10, 0.36)
Marital status	0.26 (-0.04, 0.56)	0.07 (-0.24, 0.37)
Number of children	0.21 (-0.18, 0.59)	**-0.67 (-1.05, -0.29)**
Area	**0.96 (0.14, 1.78)**	-0.85 (-1.63, 0.07)
Age* Area	0.02 (-0.11, 0.16)	-0.03 (-0.16, 0.09)
Gender* Area	-0.10 (-0.26, 0.07)	0.09 (-0.06, 0.25)
Education * Area	-0.11 (-0.28, 0.07)	-0.09 (-0.25, 0.08)
Occupation * Area	0.08 (-0.04, 0.20)	0.06 (-0.06, 0.17)
Religion * Area	-0.10 (-1.51, 1.31)	**0.24 (0.001, 0.47)**
Marital status * Area	-0.15 (-0.41, 0.12)	-0.19 (-0.44, 0.05)
Number of children*Area	-0.08 (-0.32, 0.15)	**0.33 (0.09, 0.57)**

Discussion

The overall rate of full immunization among children in Mysore, India, at 52.2%, was low. It was significantly predicted directly by parental perceptions about benefits/facilitators to childhood vaccination and indirectly predicted by parental education and employment status. Place of residence moderated the effect of religion and the 'number of children' on parental perceptions about barriers to childhood vaccination.

The full childhood vaccination rate reported by urban parents was 62.3% compared to a reported rate of 42.6% in rural schools. A pooled analysis of 108,057 Indian children aged 12–23 months during a national survey for the period 2007–08 reported similar rates of full childhood vaccination of 65.6% for urban vs. 53.6% for rural residents [12]. Analysis of the National Family Health Survey data collected during 1992–93, 1998–99, 2005–06 and 2015–2016 also showed a lower vaccination rate among rural Indian children compared to those living in urban areas [9, 42, 43]. Low education and lack of awareness about the importance of childhood vaccines, poor access to healthcare and vaccines, may at least partially explain the lower rural vaccination rates [8,9]. Indeed, when compared to urban parents (14.9%), a significantly greater portion of parents living in rural areas were illiterate (63.4%).

Parents perceiving that childhood vaccination was beneficial (e.g. effective in preventing disease, ensure their child health, help protect other children); those who felt responsible if anything bad happened did not have their child vaccinated, and those who heard about the vaccine or received a positive recommendation from a doctor or nurse were more likely to report full vaccination. In addition, parental attitudes about childhood vaccination played a mediating role between sociodemographic characteristics and the ‘full vaccination’ rate and this conquered with a previous report [44]. A number of studies in India, Pakistan, Nigeria, Benin and Uganda reported similar results for the relationship between levels of vaccination and parental attitudes and beliefs related to immunization [45,46]. Similarly, a UK study showed that the need to protect children and help protect others through herd immunity was seen to influence parental decisions about vaccinations [47] positively.

These findings indicate key issues in the perceived benefits domain of HBT that could be leveraged to improve childhood vaccination rates. Community-based education campaigns

Page: 35

Number: 1

Reiterating here the earlier comment about literacy

focused on increasing parental awareness about the benefits of childhood vaccination in preventing disease and promoting child health may be useful. In addition, strategies led by physicians and nurses should focus on strong childhood vaccination recommendations. The psychological decision-making frameworks suggested that when individuals are uncertain, they are more open to information about vaccination in their decision-making [48]. This study also suggests that intervention to increase vaccination in children may target change in parental perception about the benefits of vaccination in protecting others, which agree with the vaccine decision-making framework [47].

Full immunization rate was also associated with the educational status of the parents. Studies in different regions of India also confirmed the correlation of full vaccination with an increased educational status of the parents [12, 17-19, 21, 24,49,50]. As education level increases, awareness of disease susceptibility and severity and knowledge about the importance of vaccines increases. A study in India showed that health knowledge and the ability to communicate mediated the relationship between maternal education and childhood immunization decisions [51]. It is also likely that educated parents have better access to health information, health infrastructure, and vaccination services, further facilitating decisions about childhood vaccination.

Some of the strengths of this study include having a reasonably large sample, high response rate, and probability sampling. [2]n addition, unlike [1]~~ost~~ many studies in India [12-26], did not use as this study did, a health theory-driven model with robust analytical techniques (SEM). The study also ha limitations. The chi-square test of Model Fit for the Proposed Model (Fig 1) was significant, suggesting that the covariance matrix of the proposed model and the observed data were different. The chi-square test was, however, sensitive to sample size and

Page: 36

Number: 1

Number: 2

Suggest revising this sentence for clarity

approximation. Thus, the proposed model is acceptable for explaining the data. In addition, other model fit statistics, including RMSEA, CFI, and TLI values, indicated the fit of the proposed model to the current data. Moreover, mediating effects need to be interpreted in the context of a cross-sectional study. Furthermore, vaccination status for the children was reported at a family level. As there could be differential immunization status among children in a family, full immunization rates might have been falsely lowered. However, after stratifying data based on the number of children, full immunization rates remained similar between parents with one child versus those with two or more. The number of children was also not associated with the full immunization rate in the SEM. It is also possible that data was influenced by recall bias; parents may not have correctly reported the vaccination status of children, Due to the self-reported nature of the data, parents may also have ~~been~~ answered questions differently for reasons of social desirability. Finally, we are reporting on data collected from parents who had adolescent daughters attending school during the study period. As a result, the generalizability of the current results might be limited to parents with at least one school-going adolescent daughter. The generalizability of the study findings to parents without school-going adolescent girls or with only school-going boys will be further limited due to gender biases in immunization in India [52,53]. It is also possible that there may be recall bias and parental attitudes may have changed about childhood vaccinations. Thus, policy measures designed to change negative attitudes and beliefs of parents about childhood vaccination based on the current results should be done carefully considering potential changes over the period of time. In addition, the current HBT derived framework may not be fully applicable to explain the predictors of childhood vaccination in the Indian population.

Number: 1

And simultaneously controlling for parent age correct?

Number: 2

Number: 3

One would expect the overall reported vaccination rates to have been higher if this was the case correct?

Number: 4

Reiterating here the earlier point about the need to explain more clearly why only girls were chosen

Conclusions

In conclusion, full immunization rates were relatively low among children in Mysore, India. Misperceptions about the benefits of routine vaccination, lack of parental education and employment were barriers to receiving routine childhood vaccinations. Interventions are needed to improve parental awareness about the benefits of childhood vaccination, particularly among uneducated parents in rural areas. There may be other supply-related factors that require structural intervention to ensure that information and immunization are more widely available, particularly in rural areas. Future studies on supply-related factors are needed. The current study confirmed complementary relationships among sociodemographic, environment and attitudinal factors in predicting childhood vaccination based on HBT. Additional longitudinal studies are needed to confirm these relationships.

Acknowledgments

This study was funded by an Investigator-Initiated Award from Merck & Co., Inc. awarded to PM. KK was funded as a US Scholar by Fogarty International Center and National Heart Lung and Blood Institute, and National Institute of Neurological Disorders and Stroke and of the National Institutes of Health under Award Number D43 TW010540. PM was partially funded by grants from National Institutes for Health (D43-TW010540; R15-AI128714). The funder had no role in the study design, data collection, analysis, interpretation, or publication of the manuscript. We would like to thank the study participants for taking the time to complete the questionnaires. We would like to thank also the Block Development Officer for Mysore and the administrative staff of the schools for their assistance during data collection.

Number: 1

Were any important or interesting outcomes observable or observed with respect to religion? Even if not reliably evaluable readers will be looking for some consideration of this topic.

Competing interest

The authors reported no conflict of interest. The funder does not alter our adherence to PLOS ONE policies on sharing data and materials.

References

1. WHO: Immunization coverage. https://www.who.int/news-room/fact-sheets/detail/immunization-coverage. Accessed September 29, 2019.
2. Keja K, Chan C, Hayden G, Henderson RH: Expanded programme on immunization. https://www.ncbi.nlm.nih.gov/pubmed/3176515. World Health Stat Q. 1988;41(2):59-63.
3. WHO: Immunization, Vaccines and Biologicals. https://www.who.int/immunization/newsroom/press/immunization_coverage_july2014/en/. Accessed September 29, 2019.
4. UN: Inter-agency Group for Child. Levels & Trends in Child Mortality. 2013
5. Madhavi Y: Vaccine policy in India. PLoS Med. 2005; 2 (5): e127.
6. Oldfield BJ, Stewart RW: Common Misconceptions, Advancements, and Updates in Pediatric Vaccination Administration. South Med J. 2016;109(1):38-41.
7. Gurnani V, Haldar P, Aggarwal MK, et al: Improving vaccination coverage in India: lessons from Intensified Mission Indradhanush, a cross-sectoral systems strengthening strategy. BMJ., 2018;363.
8. Laxminarayan R, Ganguly NK: India’s vaccine deficit: why more than half of Indian children are not fully immunized, and what can—and should—be done. Health Aff. (Millwood), 2011;30:1096–103.
9. Singh PK: Trends in child immunization across geographical regions in India: focus on urban-rural and gender differentials. PLoS One. 2013; 8(9):e73102.

10. Francis MR, Nohynek H, Larson H, et al: Factors associated with routine childhood vaccine uptake and reasons for non-vaccination in India: 1998-2008. Vaccine. 2018;36(44):6559-6566.
11. Choudhary M, Solomon R, Awale J, et al: Demand-side determinants of timely vaccination of oral polio vaccine in social mobilization network areas of CORE Group polio project in Uttar Pradesh, India. BMC Infect Dis. 2018;18(1):222.
12. Shrivastwa N, Gillespie BW, Kolenic GE, et al: Predictors of vaccination in India for children aged 12-36 months. Vaccine. 2015;33:D99-105.
13. Som S, Pal M, Chakrabarty S, Bharati P: Socioeconomic impact on child immunization in the districts of West Bengal, India. Singapore Med J. 2010;51(5):406-12.
14. Shrivastwa N, Wagner AL, Boulton M: Analysis of State-Specific Differences in Childhood Vaccination Coverage in Rural India. Vaccines. (Basel) 2019; 24:7(1)
15. Rammohan A, Awofeso N: District-level variations in childhood immunizations in India: the role of socio-economic factors and health infrastructure. Soc Sci Med. 2015; 145:163–172.
16. Rathi A, Meena G: Demand and supply side factors affecting utilization of immunization Services in an Urban Village of Delhi. Adv Tech Biol Med. 2017;5:1.
17. Devasenapathy N, Jerath SG, Sharma S, et al: Determinants of childhood immunization coverage in urban poor settlements of Delhi, India: a cross-sectional study. BMJ Open. 2016;6:e013015.
18. Awasthi A, Pandey CMM, Singh U, et al: Maternal determinants of immunization status of children aged 12–23 months in urban slums of Varanasi, India. Clin Epidemiol Glob Heal. 2015; 3:110–116.

19. Ghosh A, Laxminarayan: Demand- and supply-side determinants of diphtheria-pertussis-tetanus non vaccination and dropout in rural India. Vaccine. 2017;35:1087–93.
20. Gupta P, Prakash D, Srivastava JP: Determinants of immunization coverage in Lucknow district. N Am J Med Sci. 2015; 7:36–40.
21. Kumar S, Prabhu S, Jose AP, et al: Assessment of under-five immunization coverage among the population of slum areas in Mangalore taluk, India. Int J Community Med Public Heal. 2017;4:781.
22. Singhal G, Mathur H, Dixit M, et al: Factors affecting immunization among children of rural population of block Malpura, district Tonk, Rajasthan, India. Int J Community Med Public Heal. 2016;3:641–6.
23. Srivastava AK, Shankar G: A study of immunization coverage and its determinants among under five children residing in urban field practice area of S. N. Medical college, Bagalkot, Karnataka, India. Indian J Forensic Community Med. 2017;4:48–52.
24. Mukherjee S, Madhivanan P, Li T, et al: Correlates of completing routine vaccination among children in Mysore, India. J Infect Public Health. 2015;8:62–71.
25. Eberhardt MS, Pamuk ER: The importance of place of residence: examining health in rural and non-rural areas. Am J Public Health. 2004; 94 (10): 1682–1686.
26. Das D, Pathak M: The growing rural-urban disparity in India: some issues. Int J Adv Res Tech. (2012): 1-7.
27. Montaño DE, Kasprzyk D: Theory of reasoned action, theory of planned behavior, and the integrated behavioral model. In: Glanz K, Rimer BK, Viswanath K (Eds.), Health behavior and health education: theory, research, and practice, 4th ed. San Francisco: Jossey-Bass. 2008, p. 67-96.

28. Jones CL, Jensen JD, Scherr CL, et al: The Health Belief Model as an explanatory framework in communication research: exploring parallel, serial, and moderated mediation. Health Commun. 2015;30(6):566-76.
29. Coe AB, Gatewood SBS, Moczygemba LR, Goode JKR, Beckner JO: The use of the health belief model to assess predictors of intent to receive the novel (2009) H1N1 influenza vaccine. Innov Pharm. 2012;3(2):1-11.
30. Cheney MK, Robert John R: Underutilization of Influenza Vaccine: A Test of the Health Belief Model. SAGE Open; 2013: 1–12.
31. Gerend MA, Shepherd JE: Predicting human papillomavirus vaccine uptake in young adult women: Comparing the Health Belief Model and Theory of Planned Behavior. Ann Behav Med. 2012; 44(2): 171-180.
32. Census; 2011. http://censusindia.gov.in/2011-Common/CensusData2011.html. Accessed Sept 30, 2019.
33. Degarege A, Krupp K, Fennie K, et al: An integrative behavior theory derived model to assess factors affecting HPV vaccine acceptance using structural equation modeling. Vaccine. 2019;37(7):945-955.
34. Degarege A, Krupp K, Fennie K, et al.: Urban-Rural Inequities in the Parental Attitudes and Beliefs Towards Human Papillomavirus Infection, Cervical Cancer, and Human Papillomavirus Vaccine in Mysore, India. J Pediatr Adolesc Gynecol. 2018;31(5):494-502.
35. Degarege A, Krupp K, Fennie K, et al.: HPV vaccine acceptability among parents of adolescent girls in a rural area Mysore, India. J Pediatr Adolesc Gynecol. 2018;31(6):583-591.

36. Muthén LK, Muthén BO: Mplus User's Guide. Eighth Edition (1998-2017). Los Angeles, CA: Muthén and Muthén.

37. Hair JF, Tatham RL, Anderson RE, Black W: Multivariate data analysis. (Fifth Ed.) 1998 Prentice-Hall:London.

38. Hu L, Bentler PM: Cutoff criteria for fit indexes in covariance structure analysis: Conventional criteria versus new alternatives. Structural Equation Modeling. 1999; 6(1): 1-55.

39. Byrne BM: Structural Equation Modeling with Mplus: Basic Concepts, Applications and Programming. 2011. Routledge Academic, New York.

40. Muthe´n BO, du Toit SHC, Spisic D: Robust inference using weighted least squares and quadratic estimating equations in latent variable modeling with categorical and continuous outcomes. Retrieved from www.statmodel.com/bmuthen/articles/Article_075.pdf [Accessed April 2018].

41. Tenko Raykov T, Marcoulides GA: Using the Delta Method for Approximate Interval Estimation of Parameter Functions in SEM. Struct Equ Model. 2004; 11:4, 621-637.

42. Prusty RK, Kumar A: Socioeconomic dynamics of gender disparity in childhood immunization in India, 1992-2006. PLoS One. 2014;9(8):e104598.

43. Ministry of Health and Family Welfare. Indian fact sheet: National Family Health Survey (NFHS-4) Accessed from http://rchiips.org/NFHS/pdf/NFHS4/India.pdf in July 6 2020.

44. Prislin R, Dyer JA, Blakely CH, et al.: Immunization status and sociodemographic characteristics: the mediating role of beliefs, attitudes, and perceived control. Am J Public Health. 1998;88(12):1821-6.

45. Rainey JJ, Watkins M, Ryman TK, et al.: Reasons related to non-vaccination and under-vaccination of children in low and middle income countries: findings from a systematic review of the published literature, 1999-2009. Vaccine. 2011;29(46):8215-21.

46. Vonasek BJ, Bajunirwe F, Jacobson LE, et al: Do Maternal Knowledge and Attitudes towards Childhood Immunizations in Rural Uganda Correlate with Complete Childhood Vaccination? PLoS One. 2016;11(2):e0150131.

47. Skea ZC, Entwistle VA, Watt I, Russell E: Avoiding harm to others' considerations in relation to parental measles, mumps and rubella (MMR) vaccination discussions–an analysis of an online chat forum. Soc Sci Med. 2008;67(9):1382–90.

48. Chaudry A, Henly J, Meyers M: Conceptual Frameworks for Child Care Decision-Making. White Paper. Administration for Children & Families 2010. Accessed from https://www.acf.hhs.gov/sites/default/files/opre/conceptual_frameworks.pdf, on July 7, 2020.

49. Holipah, Maharani A, Kuroda Y: Determinants of immunization status among 12- to 23-month-old children in Indonesia (2008-2013): a multilevel analysis. BMC Public Health. 2018;18(1):288.

50. Khan J, Shil A, Prakash R: Exploring the spatial heterogeneity in different doses of vaccination coverage in India. PLoS One. 2018;13(11):e0207209.

51. Prislin R, Dyer JA, Blakely CH, et al: Immunization status and sociodemographic characteristics: the mediating role of beliefs, attitudes, and perceived control. Am J Public Health. 1998; 88(12): 1821–1826.

52. Prusty RK, Kumar A: Socioeconomic Dynamics of Gender Disparity in Childhood Immunization in India, 1992–2006. Plos One 2014; 9(8): e104598

53. Corsi DJ, Bassani DG, Kumar R, et al: Gender Inequity and Age-Appropriate Immunization Coverage in India From 1992 to 2006. BMC Int Health Hum Rights. 2009;9 (Suppl 1):S3.

Fig1

Click here to access/download;Figure;Fig 1.tif

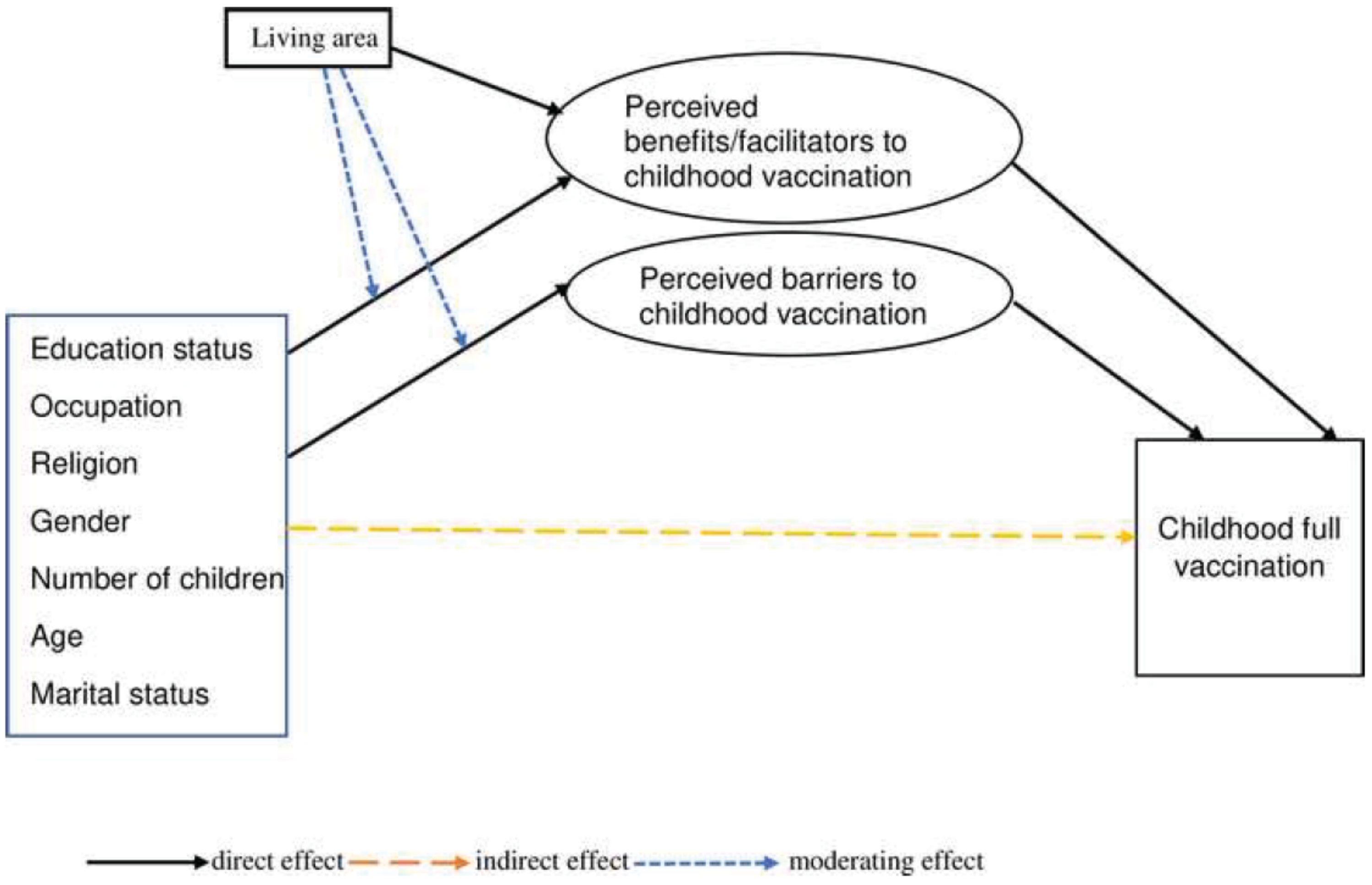

Fig2

Click here to access/download;Figure;Fig 2.tif

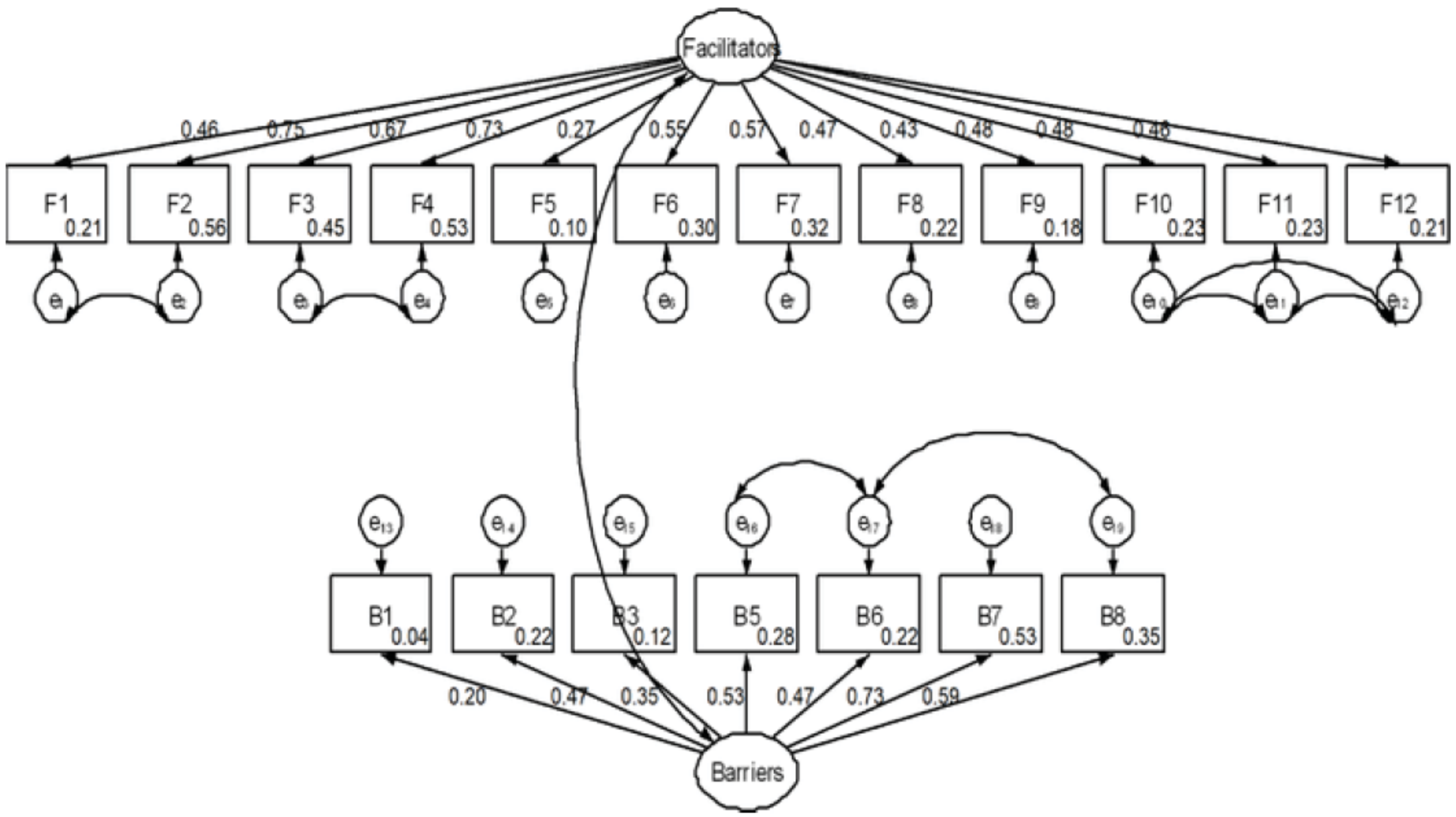

Fig3

Click here to access/download;Figure;Fig 3.tif

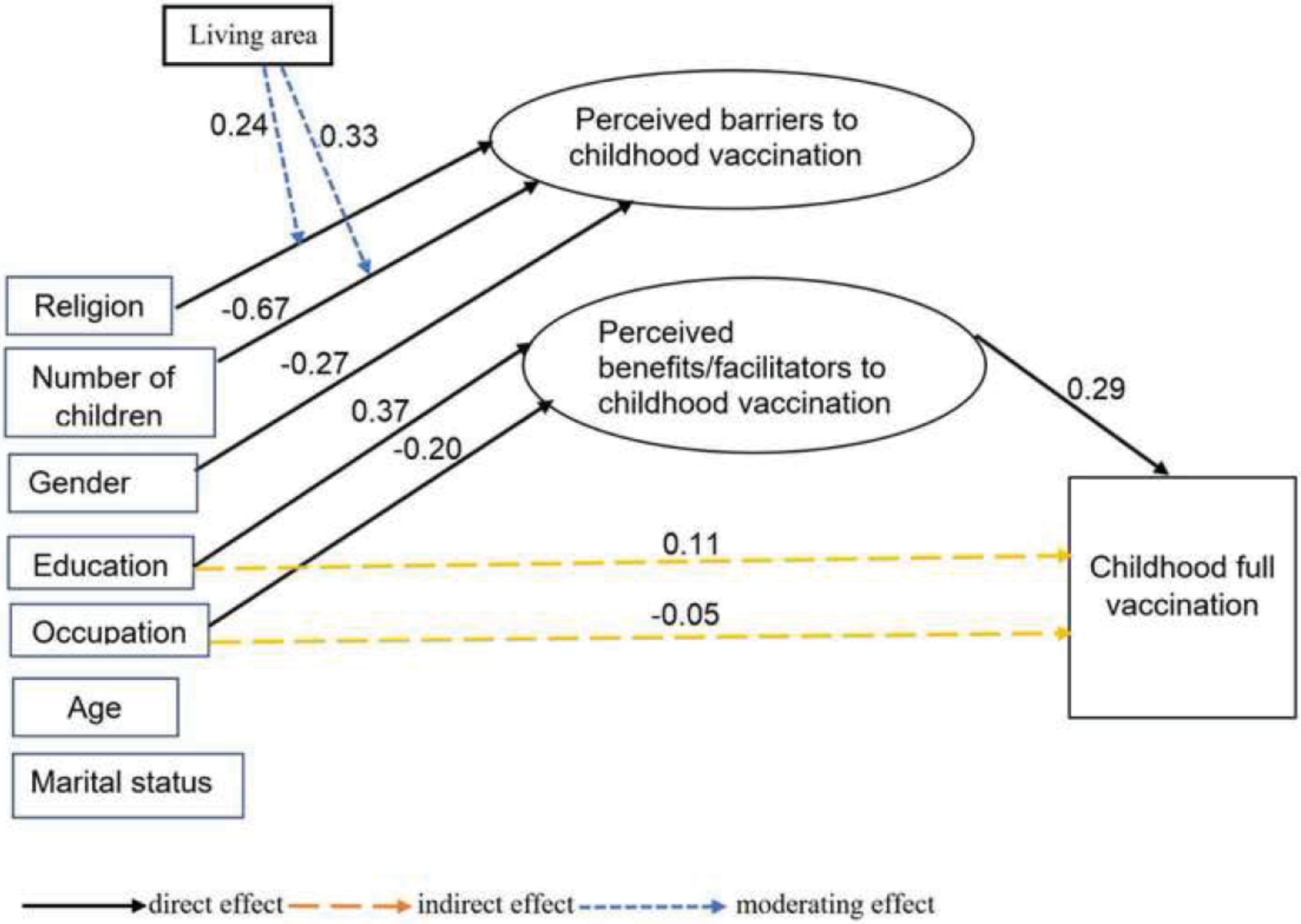

Revised Manuscript with Track Changes

Structural equation modeling to detect ~~Using Health Belief Theory derived framework to examine factors correlated with~~correlates of childhood vaccination: a moderated mediation analysis ~~using a structural equation model~~

Abraham Degarege[1]; Karl Krupp[2,3]; Vijaya Srinivas[3]; Boubakari Ibrahimou[4]; Purnima Madhivanan[2,3,5,6]

[1] Department of Epidemiology, College of Public Health, University of Nebraska Medical Center, Omaha, Nebraska, USA.

[2] Department of Health Promotion Sciences, Mel & Enid Zuckerman College of Public Health, The University of Arizona, Tucson, Arizona, USA

[3] Public Health Research Institute of India, Mysore, Karnataka, India

[4] Department of Biostatistics, Robert Stempel College of Public Health, Florida International University, Miami, Florida, USA.

[5] Division of Infectious Diseases, College of Medicine, The University of Arizona, Tucson, Arizona, USA

[6] Department of Family & Community Medicine, College of Medicine, The University of Arizona, Tucson, Arizona, USA

Corresponding Author

Abraham Degarege, MS, PhD

Assistant Professor

Department of Epidemiology, University of Nebraska Medical Center
Email: abraham.mengist@unmc.edu Phone: (1) 402-552-6682

Abstract

Objectives

This study applied used a Health Belief Theory derived framework using and Structural Equation Model to examine moderators, mediators and direct and indirect predictors of childhood vaccination.

Methods

A cross-sectional surveystudy was conducted among a random sample of 1599 parents living in urban and rural areas of Mysore district, India. Applying two-stage probability proportionate-to-size sampling, adolescent girls attending 7th through 10th grades in public, private and religious schools located in the district were selected to take home a questionnaire to their parents. A validated questionnaireParents were asked was used to assess whether their children had received at least one the routine vaccines for one dose of bacillus Calmette–Guérin; three doses of Diphtheria, Pertussis, Tetanus; three doses of oral polio vaccine; and one dose of measles-containing vaccine. In addition, parents were also asked about their attitudes towards childhood vaccination.

Results

Out of the 1599 parents, 52.2% reported that their children had received all the routine vaccines (fully vaccinated); 42.7% reported their children had missed at least one routine vaccine, and

Formatted: Font: 12 pt, Superscript

Formatted: Font: 12 pt, Superscript

5.2% reported ~~their children~~that their children had missed all routine vacc~~in~~in~~in~~ations~~-es~~. Perceptions about the benefits/facilitators to childhood vaccination significantly predicted the ~~increased~~ full vaccination rate (standardized regression coefficient (β) =0.29) directly and mediated the effect of parental education (β =0.11) and employment (β = -0.06) on the rate of full vaccination. Parental education was significantly associated ~~with higher rates of~~ indirectly with higher rates ~~an increased rate~~ of full ~~childhood~~ vaccination (β =0.11). Parental employment was significantly associated indirectly with ~~a~~ decreasing~~ed~~ rates of full vaccination (β = -0.05). Area of residence moderated the role of religion (β = 0.24) and the ‘ ~~the~~ number of children’ in a family (β =0.33) on parental perceptions about barriers to childhood vaccination. ~~M~~The model to data ~~Fit~~ ~~The fit~~ ~~of the model~~ fit was acceptable (Root Mean Square Error of Approximation = 0.02, 95% CI 0.018 to 0.023; Comparative Fit Index = 0.92; Tucker–Lewis Index = 0.91)~~to the data was acceptable~~.

Conclusions

~~Full~~ Full vaccination rate was relatively low ~~rare~~ among children in Mysore, especially ~~in those whose~~among parents who were unsure a~~hesitant a~~bout the benefits of routine vaccination and those with low educational levels~~were uneducated~~. Interventions ~~that can increase~~increasing awareness of ~~about~~ the benefits of childhood vaccination ~~may help to improve full vaccination rates in Mysore. The interventions should~~ that target ~~targeting~~ ~~uneducated~~ ~~rural~~ rural parents with lower levels of education ~~particularly those living in rural area.~~may help increase the rate of ~~improve~~ full childhood vaccination- in India~~rates in Mysore~~.

Keywords: Childhood vaccination, Structural equation model, Attitude, Parents, India

Introduction

Childhood i~~I~~mmunization ~~is~~ remains one of the most cost-effective and widely used strategies to reduce morbidity and mortality from~~in children due~~ ~~to vaccine~~ vaccine-preventable diseases. ~~Currently, the lives of~~Annually, vaccination prevents the death of two to three million children ~~are saved due to immunization~~worldwide [1]. More child deaths ~~of children~~can be prevented ~~can be averted~~ by further improving vaccination levels, ~~of vaccination~~ coverage, and timeliness ~~of the vaccines' administration~~ [1]. In order to reduce childhood mortality, the World Health Organization (WHO) launched the Expanded Program on Immunization (EPI) in 1974 with the goal of increasing ~~that aimed to develop and expand~~ immunization ~~all over the~~ worldwide [2]. In 2012, the WHO ~~, through the EPI program,~~ proposed ~~the~~ ambitious goals to immunize 90% of infants ~~in the world by~~with three doses of diphtheria-tetanus-pertussis (DTP3) vaccine by the year 2020 [3]. The ~~However, the~~ goal ~~is far from being achieved by~~remains aspirational in many developing countries [1] ~~as of now~~.~~1~~ As of~~In~~ 2018, ~~about~~ roughly 19.8 million children ~~in the world~~ did not receive routine childh~~o~~ood vaccines [1].

~~When compared to other countries in the world,~~ India has the ~~biggest~~world's largest number of annual births and ~~cohort, but~~ the lowest childhood immunization rate [4]. While India has made large strides in ~~Despite great efforts by programs including the EPI, Universal Immunization Program (UIP) and National Immunization Programme (NIP) to improve~~ immunization ~~among children in India for the~~during the ~~past 30 years~~last three decades, ~~the coverage~~immunization levels remain~~s~~ below the WHO goal of ~~low and far to reach the WHO goal of~~ ≥90% coverage for DTP3 vaccines by 2020 [3]. ~~The UIP in~~ India's Universal Vaccination Program (UIP) provides one dose of Bacillus Calmette–Guérin (BCG~~);~~), three doses of DPT3~~;~~, three doses of oral polio vaccine (OPV~~);~~), and one dose of measles-containing

vaccine (MCV) to all infants younger than one year for free [5]. Indian children ~~who~~ ~~receive~~rec~~iei~~iving all ~~these~~ four routine vaccines at ~~on~~ ~~appropriate~~ recommended doses ~~as recommended by UIP~~ are considered fully vaccinated by WHO. Children ~~who~~ receiving~~e~~ fewer than recommended doses or missing any of the childhood ~~recommended~~ vaccines are categorized as ‘under-vaccinated/partially vaccinated’, and those ~~who do not receive~~ not receiving any vaccinations are considered ‘non-vaccinated’.

Incomplete or delayed vaccination against childhood diseases can lead to increased mortality and morbidity in children [6]. Reducing ~~In order to reduce childhood mortality~~child deaths through immunization~~interrupting transmission of a vaccine vaccine preventable disease in a community,~~ ~~an~~ requires that adequate numbers -of children ~~should~~ receive full vaccination in a timely manner ~~until herd immunity is achieved~~ [6]. As of 2016, o~~However, o~~nly about 62.0% of children aged 12-23 months ~~in India~~ were fully vaccinated in India ~~in 2016, contributing to the increased child mortality rate in the country~~ [7]. India has ~~is a~~ a large and heterogeneous population with~~country so diffe~~varied ~~rences in~~ cultures. ~~Due to different~~ sociodemographics, religion~~cultural and economic reasons~~, and education status, leading to variations in vacci~~o~~nation levels among regions ~~the full immunization coverage in rural areas may particularly be lower than the national coverage for the country~~ [8,9,~~]~~10,11]. ~~demand-related factors (e.g. socioeconomic status, attitudes about childhood vaccination) might have additionally contributed to the low childhood immunization rate in the country.~~ The presence of ~~these~~ ~~under~~ under-vaccinated groups in some regions may ~~act as a~~be a -source of infectious disease ~~infection~~ ~~for~~outbreaks ~~in~~ in ~~some areas~~the country~~the community~~. ~~Therefore, it is imperative to know the level of full immunization coverage in a population to identify those that need vaccination and for planning of immunization programs accordingly. Information about full immunization~~

Formatted: Underline

~~coverage will also be useful to predict the occurrence of vaccine preventable disease epidemics in the region. Furthermore, identifying factors associated with incomplete vaccination or non-vaccination of children in the region will help plan strategies to improve full immunization rates.~~

~~Despite supply~~ supply ~~related factors including poor access to immunization service and vaccines, growth rate, population size, geographic and cultural diversity might have caused the low childhood immunization rate among children in India [10,11],~~

~~demand~~ demand ~~related factors (e.g. socioeconomic status, attitudes about childhood vaccination) might have additionally contributed to the low childhood immunization rate in the country.~~ ~~Several~~ Previous s~~s~~tudies in India have examined demand/~~individual~~ individual-related factors ~~that can affect~~affecting routine vaccination [12-26]. M~~However, m~~ost of these studies, howev~~r~~er, assumed ~~a~~ direct relationships between sociodemographic, environmental, psychological factors and childhood vaccination ~~and explored these relationships using~~ in logistic regression models [12-26]. On the other hand, some predictors of childhood vaccination may have a direct effect, and some may have an indirect effect. Furthermore, some may play- a mediating or moderating role between the predictors and childhood vaccination [27,28].

~~Indeed, health behavior theories including~~Evidence~~-~~-based on ~~Integrated Behavior Theory (IBT), Theory of Reasoned Action, Theory of Planned Behavior, and~~ Health Belief Theory (HBT) suggests~~ted~~ that factors that affect preventive behaviors such as vaccination are complex and multifaceted, and ~~will not act~~do not alway~~s~~s act in ~~a~~ similar manners [27,28]. ~~For example, the~~ According to HBT, ~~indicated that~~ individual~~s~~ perceptions about the benefits and barriers to ~~a~~ behavior ~~will~~ would directly affect the practice of ~~the~~ behavior~~,~~; ~~but~~ the effect of sociodemographic status on ~~the practice of a~~ behavior, on the other hand, would~~will~~ be indirect, ~~by~~ influencing individuals~~'~~ perceptions about the benefits and barriers ~~to~~ of ~~a~~ behavior [27,28].

In addition, as the sociodemographics status of individuals may vary between urban and rural residents in India, [8,9], we hypothesized that the effect of sociodemographic factors may vary by place of on individuals' perceptions about the benefits and barriers to a behavior may not be similar in urban and rural areasresidence (urban and rural). Despite the availability of of these health frameworks that can help us examine factors affecting that affect childhood vaccinations in a comprehensive manner, most studies on childhood vaccination in India did not use fail to do it in the context of a in India didn't examine the applicability of these theoretical framework s [12-26]. Moreover, some predictors of childhood vaccination can be directly observed, and some couldas are some be latent variables or constructs measured by a number of indicators [27,28]. As a result, methods including standard logistic regression, factor analysis, simultaneous equation modeling and path analysis may not alwayss be appropriate to model predictors offor predictors ofing childhood vaccination and exploring the explain the relationships between the predictors effectively. Health theory theory-driven complex models that employ a robust analytic technique (e.g. Structural Equation Model) are needed to better better explainexplain -the nature of the relationship between sociodemographic, attitudinal and environmental factors, while predicting correlates of routine childhood vaccination.

HBT has been used to explain the predictors of vaccination uptake related to influenza and HPV in different populations [29-31]. According to the HBT, six factors including perceived susceptibility to disease, perceived severity of a disease, expected benefits of immunization, concerns or cost associated with immunization, strategies or information sources/media that promote immunization and confidence to adopt/accept immunization would likely affect the chance of getting a vaccine [28]. Children will likely be vaccinated if parents perceive that their children are susceptible to severe diseases, believe thatin the benefit their children would get

~~t~~receive through immunization (e.g., reduce susceptibility to severe diseases) outweighs the cost/concerns ~~to get~~about the vaccine, and ~~if~~ children ~~are confident in their ability to obtain a~~appear open to receiving the vaccination [28]. ~~Although~~ HBT has, however, been mostly used as a framework for explaining vaccination~~n~~ ~~applied to explain different health behaviors, including vaccination, the theory was developed and tested mainly~~ in Western countries. In addition, the theory lacks the socio-cultural factors which could affect individuals' beliefs and behaviors related to ~~a health problem~~immunization. ~~As a result, HBT may not apply to populations with different cultures in a similar manner. However, there~~There remains ~~is~~ limited information on the utility of the HBT ~~in understanding~~ to explain parental attitudes and beliefs about childhood vaccination in ~~most~~ developing countries like ~~, including~~ India.

~~Therefore, it is imperative to~~ K~~k~~nowing sociodemographic, environmental and psychosocial ~~the level of full immunization coverage in a population to identify those that need vaccination and for planning of immunization programs accordingly. Information about full immunization coverage will also be useful to predict the occurrence of vaccine preventable disease epidemics in the region. Furthermore, identifying~~ correlates of childhood vaccination ~~factors associated with incomplete vaccination or non-vaccination of children in the region~~ will inform~~-~~ the choice of~~help plan~~ interventions ~~strategies~~ to reduce parental ~~resistance~~hesitancy to childhood vaccination~~improve full immunization~~ ~~rates.~~

The objectives of this study were~~In order to~~ to i) determine~~better understand~~ the direct and indirect predictors of childhood vaccination, ii) examine the mediating role of parental attitudes about childhood vaccination on the relationship between sociodemographic factors and childhood vaccination, iii) test the moderating role of the ~~the~~ area of residence ~~where the parents live~~ on the relationship between sociodemographic factors and parental attitudes about childhood

Formatted: No underline

Formatted: No underline

Formatted: No underline

Formatted: No underline

Formatted: No underline

Formatted: No underline

Formatted: No underline

vaccination; and iv) test the overall fit of a~~n~~ ~~Health Belief Theory~~HBT derived model ~~to~~ for analyzing childhood vaccination data.~~, we analyzed data collected on vaccine completion from urban and rural areas of Mysore District in India using Structural Equation Model (SEM).~~

Formatted: Indent: First line: 0.25

Materials and Methods

S~~tudy design and s~~tudy area

~~This was a cross-sectional study conducted among a random sample of parents of adolescent girls living in the urban and rural areas of Mysore, India.~~ The study was conducted among a random sample of parents of adolescent girls living in ~~the~~ urban and rural areas of Mysore district, India.~~, parents whose daughters aged 11 to 15 years were attending 7th through 10th grades in schools located in the urban and rural areas of Mysore district, India~~ between February 2010 and October 2011. Mysore district is in the southern part ~~of the state~~ of Karnataka, the 6th largest state by area and the 8th by population size in India. Of the 3,001,127 people living in the district (density = 450/km^2), 58.5%~~majority~~ live in rural areas (1,755,714) [32~~29~~].

Formatted: Superscript

Formatted: Superscript

Study design and ~~Study~~ participants recruitment procedure

A ~~This was a~~ cross-sectional study was conducted among a random sample of parents whose daughters aged 11 to 15 years were attending 7th through 10th grades in schools located in the urban and rural areas of Mysore D~~d~~istrict. In order to increase the chance of inclusion of parents with a ~~range~~variety of backgrounds ~~and ensure~~while ensuring the representativeness of the samples ~~to all parents with school-going adolescents girls in the district~~, two-stage probability

proportionate-to-size sampling ~~method~~ was applied to select adolescent girls~~, who were~~ attending public, private and religious schools located in the district.

Study participants recruitment procedures are ~~is~~ explained in detail elsewhere [33~~0~~,34~~1~~]. In brief, 12 schools in the urban area (five government, four private and three religious) and 11 schools in the rural areas (10 government and one private) of Mysore d~~D~~istrict were selected based on probability proportionate to size sampling. ~~Then, a~~A program announcement was sent home with all girls in~~attending~~ 7th through 10th grades in the selected schools, explaining the objectives of the study and inviting eligible parents to participate. A~~Following this, all girls aged 11 to 15 years attending grade~~ ~~7th to 10th in these schools were enumerated.~~ Of all the girl students in the selected age group in the select schools~~se girls~~, 800 girls ~~girls~~ from urban schools, and 850 girls ~~girls~~ from rural schools were randomly selected and provided with a questionnaire and consent form to take home to their ~~their~~ parents. The g~~G~~irls were expected to return~~ed~~ the completed questionnaire and ~~the~~ signed a consent form within a week ~~within seven days~~. Only one parent ~~in each family~~ was required to complete the questionnaire. A small proportion of parents in the urban (2.7%) and rural (2.2%) areas did not return the completed questionnaire and/or ~~the~~ signed the~~ed~~ consent form. IRB approval for this study was obtained from Florida International University and Public Health Research Institute of India~~, and parents provided written informed consent~~.

Formatted: Indent: First line: 0.5

Questionnaire

The questionnaire was administered in *Kannada*, the local language, ~~primarily~~ to assess factors ~~that could~~ affecting general ~~vaccines~~ vaccination and ~~specifically~~ HPV vaccination in particular~~e acceptance~~. A d~~D~~etailed~~s~~ description of the ~~on the items included in the~~ questionnaire ~~are~~ is

described elsewhere [33~~0~~-35~~2~~]. ~~The questionnaire~~It included items such as “*Please indicate (to the best of your knowledge~~)~~ whether all your children have had the following recommended vaccinations*” to ~~that assess whether their children had received~~assess immunization for BCG, DPT3, OPV and MCV. Responses were recorded as four categories (0= No, 1= Not sure, 2= Yes, 3= Not applicable). ~~In addition, the questionnaire included 20~~Twenty items (Table 1) ~~to~~ assessed parental attitudes about childhood vaccination, and ~~and~~ seven items described the~~to assess the~~ sociodemographic status of ~~the~~ parents (gender, age in years, marital status, religion, occupation, educational status, number of children in a family) (Table 2). The 20 items used to examine parental attitudes about childhood vaccination were grouped into two constructs: facilitators/benefits ~~to~~ of childhood vaccination (12 items), and barriers to childhood vaccination (8 items). Responses to items ~~that assess~~assessing parental attitudes about childhood vaccination were recorded in four categories (0= No, 1= Not sure, 2= Yes, 3= Not applicable).

Table 1. Latent variables/constructs and the corresponding measuring items along with their responses

Formatted: Font: 12 pt

Formatted: Font: 12 pt

Formatted: Font: Font color: Auto

Formatted: None, Space Before: 0 pt, After: 8 pt, Line spacing: Multiple 1.08 li

Responses to all the items were recorded in four categories: 0= No, 1= Not sure, 2= Yes, 3= Not applicable.

Table 1. Latent variables/constructs and the corresponding measuring items along with their responses

Constructs		Item
Facilitators to childhood vaccination (F)	F1	Vaccination are effective in preventing disease
	F2	It is very important that my children receive all their vaccine
	F3	Vaccine is one way that parents can ensure their child health
	F4	I have a responsibility to have my children vaccinated for the protection of all children
	F5	Parents should make their decision for their child rather than a doctor
	F6	The government does a good job providing vaccine and health services
	F7	There are [illegible] vaccines included in the childhood vaccination schedule
	F8	I would feel responsible if anything bad happened I did not have my child vaccinated
	F9	I would know where to go if I wanted to have my child vaccinated
	F10	When we visit a doctor/nurse, they tell us about vaccinations for my child
	F11	When we visit a doctor/nurse, we ask about vaccination for our child
	F12	We always get the vaccinations recommended by a doctor or nurse
Barriers to vaccination (B)	B1	I am concerned about vaccine side effects
	B2	I am afraid of vaccination of my children
	B3	It is better to get the disease and protected
	B4	I would feel responsible if anything bad happened I had my child vaccinated
	B5	The high cost of transportation would affect my decision about whether to vaccinate my child
	B6	Cost is an important factor in deciding whether to vaccinate my child
	B7	Getting time off from work or household duties makes it difficult to take my child for vaccination
	B8	I would not give optional vaccines to my child because it is too expensive

Table 2. Sociodemographic characters of the study participants/parents and vaccination status of their children at a family level in Mysore, India 2010/2011

Sociodemographic Characters of the parents	Categories (n)	Vaccination status of children at a family level			p-value
		Fully vaccinated (%)	Under vaccinated (%)	No ~~Un~~vaccination~~ed~~ (%)	
Education	No formal education (636)	39.3	53.1	7.55	<0.001
	Grade 1 to 10th (676)	54.0	41.86	4.1	
	High school or above (287)	76.3	21.3	2.4	
Occupation	Unemployed (724)	54.7	40.19	5.11	0.172
	Employed (875)	50.1~~06~~	44.~~69~~7	5.~~26~~3	
Area	Rural (822)	42.6~~58~~	51.3~~4~~	6.~~08~~1	<0.001
	Urban (777)	62.3~~29~~	33.~~46~~5	4.~~25~~3	
Age (years)	≤35 (679)	47.3~~28~~	46.5~~4~~	6.~~19~~ 2	0.003
	>35 (920)	55.~~76~~8	39.~~78~~8	4.5	
Gender	Males (433)	53.6	42.7	3.7	0.248
	Females (1166)	51.6	42.6	5.8	
Religion	Hindus (1420)	51.6	43.2	5.1~~4~~	0.133
	Muslims (154)	5.1	40.9	4.6	
	Christians (25)	68.0	20.0	12.0	
Marital status	Married (1487)	52.6	42.2	5.2	0.441
	Single (112)*	46.4	48.2	5.4	
Number of children in a family	One (131)	55.0	39.7	5.3	0.773
	Two (719)	55.8	39.6	4.6	
	Three (749)	48.2	46.1	5.7	

Single=unmarried or separated or widowed

Formatted Table

Formatted: Justified, Indent: Hanging: 0.07

Formatted: Font: (Default) Times New Roman, 12 pt

Formatted: No Spacing, None, Space Before: 0 pt, After: 0 pt, Line spacing: single

Formatted: Font: 12 pt, Not Bold, Font color: Auto

Data analysis

The M plus (version 7.3) software package was used for data analysis [36~~3~~]. The main outcome variable ~~in this study~~ was family childhood vaccination status ~~in a family. Childhood vaccination status in a family was~~ grouped into three categories: fully vaccinated, ~~under~~ under-vaccinated and no ~~un~~vaccination~~ed~~. 'Full~~-ly~~ vaccination' ~~referred to all children in a~~described families with children that had~~y~~ ~~having~~ received one dose of BCG, three doses of DPT, three doses of OPV and one dose of MCV. ~~When~~ If at least one child in a family ~~did~~ had not received one or more of these vaccines, it was grouped as 'incomplete/~~under~~ under-vaccinated~~'.~~,' and ~~those who did~~if children had not received any ~~of these~~ vaccination,~~es~~ the family was ~~were~~ grouped as 'no ~~un~~vaccination~~ed'.~~.'

A modified conceptual model ~~following the~~informed by HBT was used to guide the ~~his~~ analysis (Fig 1). Rates of full vaccination ~~was~~ were predicted by parental perceptions about the benefits/facilitators and barriers to childhood vaccination (Table 1). Parental perceptions about the benefits/facilitators and barriers to childhood vaccination ~~was~~ were in turn predicted by sociodemographic variables which included gender (females/males), age group (<35/≥35 years), marital status (unmarried/married), religion (Muslims/Hindus/Christians), occupation (unemployed/employed), educational status (no formal education/formal education), and the number of children in a family (one/two or three). As ~~the~~ a number of participants ~~who~~ reported 'not applicable' to some of the items used to assess parental attitudes about childhood

vaccination were small (<20) or none, they were treated as missing during the analysis. Similarly, the number of participants who reported 'not applicable' or "not sure" ~~to the items used to assess whether all children in the family had received the recommended vaccinations~~ were also small (<10~~),~~), so they were ~~also~~ ~~; they were~~ excluded from the analysis.

Fig 1. Proposed Health Belief Theory derived model for examining factors predicting ~~childhood~~ ~~full~~full childhood vaccination.

A confirmatory factor analysis was used to check the reliability and validity of the items used for measuring the constructs of perceived benefits/facilitators and perceived barriers to childhood vaccination. The strength of the influence or correlation of the scores of the items with the scores of the constructs, (i.e. the variability in the scores of the constructs explained by the items)~~,~~ was determined based on the magnitude of the factor loadings for each item [37]. The cut-offs used were ≤ 0.32 (poor), 0.33 - 0.45 (fair), 0.46 - 0.55 (good), 0.56 - 0.69 (very good), ≥ 0.70 (excellent). ~~-~~SEM was used to examine~~check~~ ~~: i) the direct effect of~~ parental perceptions that directly predicted ~~about the benefits/facilitators and barriers to childhood vaccination on the~~ childhood ~~rate of full~~ vaccination~~,~~ and sociodemographic factors that indirectly predicted ~~-~~the behavior. SEM was also used to evaluate the ~~; ii)~~ mediating roles~~effect~~ of parental perceptions ~~about the benefits/facilitators and barriers to childhood vaccination~~ on the relationship between sociodemographic factors and ~~the rate of full~~childhood vaccination and~~; iii) total and specific indirect effects (i.e. the portion of the total indirect effect that works through perceptions about benefits /facilitators and barriers to childhood vaccination separately) of sociodemographic factors on the rate of full vaccination; iv)~~ the moderating role of ~~the area where~~ 'rural or urban residence' ~~the parents were living~~ on the relationship between sociodemographic factors ~~variables~~ and parental perceptions about ~~about the benefits and barriers to~~ childhood vaccination.

Formatted: Not Highlight

Moreover, SEM was applied to test ~~, and v)~~ model fit of the proposed HBT derived model (Fig 1) with the observed data.

M~~Overall m~~odel fit was checked using ~~chi-~~chi-square (model acceptable if *P*~~p~~ > 0.05) and other fit indices including Comparative Fit Index (model acceptable if CFI > 0.90), Tucker–Lewis Index (model acceptable if TLI > 0.90), and Root Mean Square Error of Approximation (model acceptable if RMSEA is < 0.08) [38~~4~~]. Before fitting the SEM model that has the measurement and structural components in it, fit of the measurement model that included the constructs 'perceived benefits/facilitators' and 'barriers' to childhood vaccination was checked. In order to improve the fit of the measurement model, a slight modification was made by removing items with factor loading < 2 and by allowing some of the items to freely co-vary. Similarly, we introduced slight changes in the SEM model based on the Mplus model fit indices~~s~~ outputs [39~~5~~]. Since the outcome variable (full vaccination status) was categorical~~ in nature~~, the Weighted Least Squares Estimation Method was used to estimate the parameters (path coefficients, factor loadings, variances, and covariances) and model fit statistics [40~~36~~]. Standard errors and 95% CI for direct and indirect effects were estimated using the delta method [41~~37~~].

Formatted: Font: Italic

Results

Characteristics of the study participants

Out of 1650 parents contacted, 19 from rural and 22 from ~~the~~ urban areas did not return the questionnaire and/or the signed~~ed~~ the consent form. In addition, another ten parents from ~~the~~ urban and rural areas responded to the question of uptake of routine childhood vaccines as 'not applicable' or 'not sure'. Data for the remained 1599 parents were analyzed for this study.

~~Majority~~ The majority of ~~the~~ study participants were mothers (76.7%), illiterate (63.4%), reported their religion as Hindus (99.0%), and were aged ≤35 years (53.3%). Rates of full vaccination, under vaccination and non-vaccination among children, ~~of the study participants,~~ were 52.2%, 42.7% and 5.2%, respectively. ~~Rate~~ The rate of full vaccination ~~in children~~ was significantly greater among parents who were educated (60.6%), ~~,~~ ~~among~~ urban residents, (62.3%)~~,~~ and those aged >35 years (55.8%) (Table 2).

Structural e~~E~~equation m~~M~~m~~Measurement m~~odel

The measurement model for the construct 'perceived benefits/facilitators ~~'~~ to childhood vaccination" that included all the items listed in table 1 ~~that~~ fitted acceptably to the data based on the RMSEA, ~~;~~ ~~B~~but the model showed a lack of~~se didn't~~ fit based on CFI and TLI values (RMSEA = 0.05, CFI = 0.89, TLI = 0.86~~, and β = 0.29 to 0.74~~). After ~~However,~~ allowing ~~the~~ some residual terms for ~~some~~ items to covary (F1 WITH F2; F3 WITH F4; F10 WITH F11; F10 WITH F12; F11 WITH F12) ~~improved data,~~ fit of the construct measurement model ~~for the construct~~ 'perceived benefits/facilitators~~'~~ to childhood vaccination' improved (RMSEA =0.03, CFI =0.97, TLI =0.96~~, β = 0.28 to 0.76~~). All ~~the~~ 12 items listed in T~~t~~able 1 ~~that~~ were used to measure the construct 'perceived benefits/facilitators' that significantly loaded with ~~a~~ standardized factor loadings (β) ranging from 0.27 to 0.75. The items "*It is very important that my children receive all their vaccine*" and "*I have a responsibility to have my children vaccinated for the protection of all children*" explained sufficient variance or strongly influenced/correlated (β = 0.75 and 0.73, respectively), and the item "*Parents should make health decision for their own children rather than a doctor*" explained small variance~~/~~ weakly influenced/correlated (β = 0.27) with the score for the construct variable 'perceived

Formatted: Font: (Default) Times New Roman, 12 pt

Formatted: Font: (Default) Times New Roman, 12 pt

Formatted: Font: Italic

Formatted: Font: Italic

Formatted: Font: Italic

Formatted: Font: Italic

benefits/facilitator’ to childhood vaccination. The remaining nine items moderately influenced/correlated with the score of the construct variable ‘perceived benefits/facilitator’ (β = 0.43 to 0.67).

The measurement model for the construct ‘perceived barriers~~’~~ to childhood vaccination’ fitted acceptably to the data ~~at first~~ (RMSEA = 0.06, CFI = 0.94, TLI = 0.92, β = 0.12 to 0.66), but the factor loading index for the item ‘*I would feel responsible, if anything bad happened I had my child vaccinated*’ was 0.12. After ~~removal of~~removing i~~this i~~tems with a factor loading less than two, and allowing the residual terms for some items to covary (B5 WITH B6; B6 WITH B8), data fit of the measurement model for the construct~~’~~ ‘barriers~~’~~ to childhood vaccination’ improved (RMSEA = 0.05, CFI =0.98, TLI =0.96~~, β = 0.20 to 0.70~~). S~~These s~~even items, ~~including~~ B1- B3 & B5 - B8 used to measure the construct perceived barriers significantly loaded with a standardized factor loading ranging from 0.20 to 0.73. The item “*Getting time off from work or household duties makes it difficult to take my child for vaccination*” explained sufficient variance or strongly influenced/correlated (β = 0.73) and the item “*I am concerned about vaccine side effects*” explained small variance or weakly influenced/correlated (β = 0.20) with the score for the construct variable ‘perceived barriers to childhood vaccination.’ The observed scores for the remaining four items moderately influenced/correlated with the score of the construct variable ‘perceived benefits/facilitator to childhood vaccination’ (β = 0.43 to 0.67). The item “*It is better to get the disease and protected*” showed a modest-sized positive influence/correlation (β = 0.35) with the construct variable ‘perceived benefits/facilitator to childhood vaccination.’ -The final measurement model that included ~~all the~~ 19 items used to measure ~~the~~ facilitators and barriers to childhood vaccination~~,~~ had acceptable fit the data. ~~acceptably~~ (RMSEA =0.04, 95% CI: 0.03 - 0.04; CFI =0.93, TLI =0.91) (Fig 2).

Formatted: Indent: First line: 0.5

Formatted: Font: Italic

Formatted: Font: Italic

Fig 2. Measurement model: factor loadings of items used to measure facilitators and barriers about childhood vaccination in Mysore, India 2010/2011.

~~Structural model~~

The final full SEM model with the structural and measurement components was identified. T~~In other words, t~~he total ~~(i.e.~~ number of free parameters estimated (n=130) was less than the number of model parameters ~~the number of model parameters~~ (n=630). The number of free parameters was the sum of the numbers of estimates, including factor loadings, variances of error and covariance. The number of model parameters is the number of ~~(~~unique co-variances of measured variables~~)~~ ~~and~~ estimated as the product of the ~~. The total number of free parameters estimated was 130 (factor loadings + variances of error + covariance), and the number of model parameters/unique co-variances of measured variables was 630 (~~number of measured variables (n=35) ~~and~~ ~~×~~ (the ~~(~~number of measured variables +1) divided by ~~/~~2~~)~~.

Fit of the final SEM model to the data was also acceptable based on RMSEA, CFI and TLI values (RMSEA: 0.02: 95~~0~~% CI: 0.018 to 0.023, CFI=0.92; TLI=0.91) (Fig 3). T~~However, t~~he chi-square statistic, however,~~s~~ suggested a significant difference in the covariance matrix ~~suggested by~~in the proposed model (Fig 1) and the observed data ($\chi 2$ =3914.6, DF=490, $p < 0.01$).

Fig 3. Structural Equation Model showing moderators, mediators and direct and indirect predictors of childhood vaccination in Mysore, India, 2010/2011.

Factors affecting childhood vaccination status

F~~The rate of f~~ull childhood vaccination ~~in a family~~ significantly increased with ~~an increase in~~ parental perceptions about the benefits/facilitators to childhood vaccination ~~-~~(standardized regression coefficient (β) = 0.29, ~~p~~ *P* < 0.001). In other words, ~~,~~ it increased if parents felt the ~~i.e.~~ vaccine was~~is~~ effective in preventing disease and ~~to ensure~~ensuring ~~their~~ child health, feeling a responsibility to protect their child and others, having a healthcare provider recommendation, ~~getting~~recei~~e~~ving information about childhood vaccine ~~by~~from a doctor or nurse, knowing where to go ~~to get the child vaccinated~~for vaccinations, perceiving that the government does a good job providing vaccines and health services, and having several ~~and there are many~~ vaccines included in the childhood vaccination schedule. Parental education was significantly associated indirectly with ~~an~~ increased ~~rate of~~ full ~~childhood~~ vaccination (β = 0.08, *P*~~p~~ < 0.001). Parental employment was indirectly associated with a decreased rate of full vaccination (β = - 0.05, *P* ~~p~~= 0.05). The relationship of parental education (β = 0.08, *P*~~p~~ < 0.001) and employment (β = -0.06, *P* ~~p~~= 0.045) with the rate of full vaccination were significantly mediated by ~~the~~ parental perceptions about the benefits/facilitators to childhood vaccination. P~~However, p~~arental perceptions about barriers to childhood vaccination ~~did not show~~neither showed a significant association directly with the rate of full vaccination nor mediated the relationship between sociodemographic variables including age, gender, marital status, religion, education, occupation and number of children, and the rate of full vaccination (Table 3). In addition, age, gender, marital status, religion and number of children in a family ~~didn't have any~~had no indirect relationship with the rate of full vaccination through the construct about perceptions about the benefits/facilitators (Table 3).

Table 3. Unstandardized (B) and standardized (β) effects of factors affecting full childhood vaccination for the structural model

Formatted: Font: Italic

Formatted: Font: Italic

Formatted: Font: Italic

Formatted: Font: Italic

Formatted: Font: Italic

Factors			Full childhood vaccination	
Direct			*B* (95% CI)	*β* (95% CI)
	Facilitators		**0.62 (0.46, 0.78)**	**0.29 (0.22, 0.36)**
	Barriers		-0.12 (-0.56, 0.33)	-0.02 (-0.10, 0.06)
Indirect		Mediators		
	Age	Facilitators	0.04 (-0.02, 0.09)	0.04 (-0.02, 0.09)
		Barriers	0.002 (-0.007, 0.012)	0.02 (-0.01, 0.01)
		Sum of indirect effect	0.04 (-0.02, 0.10)	0.04 (-0.02, 0.10)
	Gender	Facilitators	0.05 (-0.02, 0.13)	0.05 (-0.02, 0.13)
		Barriers	0 01 (-0 02 0 03)	0 05 (-0 02 0 03)
		Sum of indirect effect	0.06 (-0.02, 0.14)	0.12 (-0.02, 0.14)
	Education	Facilitators	**0.11 (0.05, 0.17)**	**0.08 (0.04, 0.12)**
		Barriers	0.01 (-0.01, 0.012)	0.002 (-0.01, 0.01)
		Sum of indirect effect	**0 11 (0 05 0 17)**	**0 08 (0 04 0 12)**
	Occupation	Facilitators	**-0 06 (-0 12 -0 001)**	**-0 06 (-0 12 -0 001)**
		Barriers	-0.01 (-0.02, 0.01)	-0.01 (-0.02, 0.012)
		Sum of indirect effect	**-0.06 (-0.11, 0.000)**	**-0.05 (-0.11, 0.00)**
	Religion	Facilitators	-0.07 (-1.33, 1.20)	-0.06 (-1.32, 1.19)
		Barriers	0.01 (-0.02, 0.04)	0.01 (-0.02, 0.04)
		Sum of indirect effect	-0.06 (-1.32, 1.21)	-0.06 (-1.31, 1.20)
	Marital	Facilitators	0.08 (-0.01, 0.17)	0.078 (-0.014, 0.17)
		Barriers	-0.001 (-0.01, 0.01)	-0.001(-0.01, 0.01)
		Sum of indirect effect	0.07 (-0.02, 0.17)	0.07 (-0.015, 0.17)

Factors			Full childhood vaccination	
Direct			B (95% CI)	β (95% CI)
	Facilitators		0.62 (0.46, 0.78)	0.29 (0.22, 0.36)
	Barriers		-0.12 (-0.56, 0.33)	-0.02 (-0.10, 0.06)
Indirect		Mediators		
	Age	Facilitators	0.04 (-0.02, 0.09)	0.04 (-0.02, 0.09)
		Barriers	0.002 (-0.007, 0.012)	0.02 (-0.01, 0.01)
		Sum of indirect effect	0.04 (-0.02, 0.10)	0.04 (-0.02, 0.10)
	Gender	Facilitators	0.05 (-0.02, 0.13)	0.05 (-0.02, 0.13)
		Barriers	0.01 (-0.02, 0.03)	0.05 (-0.02, 0.03)
		Sum of indirect effect	0.06 (-0.02, 0.14)	0.12 (-0.02, 0.14)
	Education	Facilitators	0.11 (0.05, 0.17)	0.08 (0.04, 0.12)
		Barriers	0.01 (-0.01, 0.012)	0.002 (-0.01, 0.01)
		Sum of indirect effect	0.11 (0.05, 0.17)	0.08 (0.04, 0.12)
	Occupation	Facilitators	-0.06 (-0.12, -0.001)	-0.06 (-0.12, -0.001)
		Barriers	-0.01 (-0.02, 0.01)	-0.01 (-0.02, 0.012)
		Sum of indirect effect	-0.06 (-0.11, 0.000)	-0.05 (-0.11, 0.00)
	Religion	Facilitators	-0.07 (-1.33, 1.20)	-0.06 (-1.32, 1.19)
		Barriers	0.01 (-0.02, 0.04)	0.01 (-0.02, 0.04)
		Sum of indirect effect	-0.06 (-1.32, 1.21)	-0.06 (-1.31, 1.20)
	Marital	Facilitators	0.08 (-0.01, 0.17)	0.078 (-0.014, 0.17)

Formatted Table

	~~Barriers~~	~~-0.001 (-0.01, 0.01)~~	~~-0.001(-0.01, 0.01)~~
	~~Sum of indirect effect~~	~~0.07 (-0.02, 0.17)~~	~~0.07 (-0.015, 0.17)~~

~~Table 3. Unstandardized (B) and standardized (β) effects of factors affecting full childhood vaccination for the structural model~~

Effect of background factors on parental perceptions about childhood vaccination

As compared to uneducated parents, ~~educated ones~~those who were educat~~ioned~~ed were more likely to perceive that childhood vaccination was beneficiary (β = 0.37, *P* ~~p~~< 0.001). Parents living in urban areas were more likely to perceive that childhood vaccination was beneficial~~ry~~ than those living in rural areas (β = 0.96, *P*~~p~~=0.022). Employed parents were also less likely to believe that childhood vaccination was beneficial~~ry~~ as compared to unemployed ones (β = -0.20, *P* ~~p~~= 0.036). Perceptions about barriers to childhood vaccination was lower among women than in men (β = -0.27, *P* ~~p~~= 0.030). When compared to parents with one child, those with two or more children were less likely to perceive that childhood vaccinations were harmful (β = -0.67, *P* ~~p~~= 0.001). ~~Geographical~~ GA ~~g~~eographical region ~~where parents were living~~ significantly moderated the effect of religion (β =0.24, *P* ~~p~~= 0.045), ~~and~~ as did ~~the~~ number of children ~~in a family~~ (β =0.330, *P* ~~p~~= 0.007) on parental perceptions about barriers to childhood vaccination. However, the nature of the relationship of parental education, gender, age and marital status with parental perceptions

- **Formatted:** Font: Italic
- **Formatted:** Font: Italic
- **Formatted:** Font: Italic
- **Formatted:** Font: Italic
- **Formatted:** Font: Italic
- **Formatted:** Font: Italic
- **Formatted:** Font: Italic
- **Formatted:** Font: Italic

about benefits/facilitators and barriers to childhood vaccination ~~didn't~~ did not however vary based on ~~the area where parents were living~~place of residence (Table 4).

Table 4. Standardized effects of sociodemographic factors and interaction terms on parental perceptions about benefits/facilitators and barriers to full childhood vaccination for the SEM

Background (exposure)	Complete childhood vaccination (outcomes)	
	Facilitators	Barriers
	β (95% CI)	*β* (95% CI)
Age	0.12 (-0.07, 0.32)	-0.12 (-0.30, 0.07)
Gender	0.18 (-0.07, 0.43)	**-0.27 (-0.52, -0.03)**
Education	**0.37 (0.19, 0.55)**	-0.12 (-0.29, 0.05)
Occupation	**-0.20 (-0.39, -0.013)**	-0.05 (-0.23, 0.13)
Religion	-0.22 (-4.51, 4.07)	-0.37(-1.10, 0.36)
Marital status	0.26 (-0.04, 0.56)	0.07 (-0.24, 0.37)
Number of children	0.21 (-0.18, 0.59)	**-0.67 (-1.05, -0.29)**
Area	**0.96 (0.14, 1.78)**	-0.85 (-1.63, 0.07)
Age* Area	0.02 (-0.11, 0.16)	-0.03 (-0.16, 0.09)
Gender* Area	-0.10 (-0.26, 0.07)	0.09 (-0.06, 0.25)
Education * Area	-0.11 (-0.28, 0.07)	-0.09 (-0.25, 0.08)
Occupation * Area	0.08 (-0.04, 0.20)	0.06 (-0.06, 0.17)
Religion * Area	-0.10 (-1.51, 1.31)	**0.24 (0.001, 0.47)**
Marital status * Area	-0.15 (-0.41, 0.12)	-0.19 (-0.44, 0.05)
Number of children*Area	-0.08 (-0.32, 0.15)	**0.33 (0.09, 0.57)**

Discussion

The overall rate of full immunization among children in Mysore, India, at ~~a family level was~~ 52.2%~~-~~, was low. It~~The rate of full vaccination~~ was significantly predicted directly by parental perceptions about benefits/facilitators to childhood vaccination~~,~~ and indirectly predicted by parental education and employment status. ~~Area The area where the parents~~Place of residence ~~lived~~ moderated the effect of religion and the ‘~~the~~ number of children’ ~~in a family~~ on parental perceptions about barriers to childhood vaccination.

The ~~current~~ full childhood vaccination rate reported by urban parents ~~in Mysore~~ was 62.3% ~~as comparable to the average rate of 62.0% for Indian children younger than one year in 2016,[7], but the~~compared to a reported rate of 42.6% in ~~was lower in the~~ rural ~~area~~schools. A pooled analysis of 108,057 ~~178,473~~ Indian children aged 12–23 months during a national survey for th~~ree consecutive~~e period~~s (1998–99, 2002–04 and~~ 2007–08~~)~~ ~~also~~ reported similar rates of ~~a~~ full childhood vaccination ~~rate~~ of 65.6~~2~~% for urban ~~residents and~~ vs. 53~~49~~.6~~4~~% for rural~~,~~ residents [12]. Analysis of the National Family Health Survey data collected during 1992–93,

Formatted: Font color: Auto

1998–99, ~~and~~ 2005–06 and 2015–2016 also ~~consistently~~ showed a lower vaccination rate among rural Indian children ~~living in the rural areas than those in~~compared to those living in ~~the~~ urban areas [9, 42~~38~~, 43]. Low education and lack of awareness about the importance~~t~~ of childhood vaccines, poor access ~~for~~ to ~~health facility~~healthcare and vaccines, may at least partially explain~~contribute to~~ the lower ~~vaccination rate in the~~ rural vaccination rates~~area~~ [8,9]. Indeed, when compared to urban parents (14.9%), a significantly greater portion of parents living in rural areas were illiterate (63.4%).

Parents ~~who perceived~~perceiving that childhood vaccination was beneficial~~ly~~ (e.g. effective in preventing disease, ensure their child health, help protect other children) ~~to their children~~; those who felt responsible if anything bad happened did not have their child vaccinated, and those who heard about the vaccine or received ~~or asked information and got~~ a positive recommendation ~~about childhood vaccination by~~from a doctor or nurse~~,~~ were more likely to report full vaccination ~~for their children were more likely to report full vaccination for their children~~. In addition, parental attitudes about childhood vaccination ~~also~~ played a mediating role between sociodemographic characteristics and the ‘~~the~~ full vaccination’ rate and this conquered with a previous report [44~~39~~]. A number of studies in India, Pakistan, Nigeria, Benin and Uganda ~~also reported~~reported similar results for the relationship between levels of vaccination and ~~association of childhood vaccination with~~ parental attitudes and beliefs related to immunization [45~~0~~,46~~1~~]. Similarly, a ~~in a study among~~ UK study showed that ~~parents,~~ the need to protect ~~their own~~ children and help protect others through herd immunity~~,~~ was seen to influence parental decisions about vaccinations [47] positively~~s about vaccinations~~.

These findings indicate key issues in ~~areas of perceptions within~~ the perceived benefits domain ~~in the~~of HBT ~~where interventions should focus on~~that could be leveraged to improve~~d~~

Formatted: Font color: Auto

Formatted: Font color: Auto

Formatted: Font color: Auto

Formatted: Font color: Auto

Formatted: Font color: Auto

Formatted: Font color: Auto

Formatted: Font color: Auto, Not Highlight

Formatted: Font color: Auto

childhood vaccination rates. Community-based education campaigns that can increasefocused on increasing parental awareness about the benefits of childhood vaccination in preventing disease and promoting child health would be importantmay be useful. In addition, strategies led by physicians and nurses to increase childhood vaccination wouldshould focus on the provision of information about the benefits of childhood vaccination and giving strong childhood vaccination recommendations. to their patients. Information provided by health care providers will greatly enforce and pressure parents to complete vaccinations for their children. Indeed, Tthe psychological decision-making frameworks suggested that when individuals are uncertain, the they are more open to information about vaccination in recent, main and familiar information that they encountered will likely influence their decision-making [48]. This study also suggests that HBT may a useful framework for informing interventions to increase vaccination in this group intervention to increase vaccination in children may target change in parental perception about the benefits of vaccination in protecting others, which agree with the vaccine decision-making framework [47].

Formatted: Font: (Default) Times New Roman, 12 pt

Full immunization rate was also associated with the educational status of the parents. SOther studies in different regions of India also confirmed the correlationthe associated of ed an increased rate of full vaccination with an an increase in the increased educational status of the mothers parents [12, 17-19, 21, 24,492,5043]. As parents' education level increases, their awareness of disease about susceptibility and severity to different infectious diseases, and and knowledge about the importance of vaccines in prevention of these disease will also likely increases; thereby increasing uptake of routine vaccines for their children. Indeed, Aa study in India showed that health knowledge and the ability to communicate, mediated the relationship between maternal education and childhood immunization decisions [5144]. It is also possible

thatlikely that educated parents may have better access to health information, health infrastructure, and vaccination services, and may be able to makefurther facilitating decisions for their children's health more confidentlyabout childhood vaccination.

Some of the strengths of this study include having a reasonably large sample, size with a high response rate, and in which study participants were recruited using probability sampling. In addition, unlike most many studies in India [12-26], did not use which examined the direct relationship between parental characteristics and attitudes to children immunization status, using a logistic regression model; , the current study analyzed data usias this study did, ng a health theory theory-driven model with robust analytical techniques (SEM). The study also ha However, the interpretation of the current findings should consider the following limitations. The chi-square test of Model Fit for the Proposed Model (Fig 1) was significant, suggesting there was evidence that the covariance matrix by of the proposed model and the observed data were different. THowever, the chi-square test was, however, is sensitive to sample size and approximation. Thus, the proposed model is still acceptable to explainfor explaining the data. In addition, other model fit statistics, including RMSEA, CFI, and TLI values, indicated the fit of the proposed model to the current data. Moreover, mediating effects need to be interpreted in the context of a cross-sectional study.- Furthermore, the parents involved in this study had one, two or three children and at least one of them was in their adolescence. Hence, the Vvaccination status for the children was reported at a family level. As there could be differential immunization status among children in a family, the full immunization rates mamong children in the current study population might have been falsely reportedlowered. However, after stratifying data based on the number of children, full immunization rates remained similar between parents with one child versus those with two or more. NThe nThe number of children parents had was also not

associated with the full immunization rate in the SEM. Moreover, It is also possible that data was influenced by recall bias; parents may not have correctly remembered reported the vaccination status of their children, which would have had happened some years before. Missed immunization reports have been associated with higher birth order in India [5245]. DFurthermore, due to the self-reported nature of the data, parents might have biased responses to somemay also have been answered questions for differently for reasons of social desirability. Recall bias may also have influenced reporting of also be at play, as parents whose children were fully vaccinated may be more likely to recall specific barriers and facilitators to childhood vaccination than parents whose children were under- or unvaccinated. Finally, we are reporting on data collected from parents who had adolescent daughters attending school during the study period. As a result, the generalizability of the current results might be limited to parents with at least one school-going adolescent daughter. The generalizability of the study findings to parents without school-going adolescent girls or with only school-going boys will be further limited due toby to gender biases in immunization in India [52,53]. Finally,I-t is also possible that there may be recall bias and parental attitudes may have changed about childhood vaccinations. there was a delay in the time the current data were collected (February 2010 and October 2011) and that we are reporting on the results. There could be changes in the Pparental attitudes and beliefs about childhood vaccination in over the past yearsmay have changed in the intervening years. Thus, policy measures designed to change negative attitudes and beliefs of parents about childhood vaccination based on the current results should be done carefully considering potential changes over the period of time. In addition, the current HBT derived framework may not be fully applicable to explain the predictors of childhood vaccination in the Indian population.

Formatted: Indent: First line: 0.5

Conclusions

In conclusion, full immunization rates were relatively low among children in Mysore, India. Misperceptions about the benefits of routine vaccination, lack of parental education and employment were barriers to ~~their children~~ receiving routine childhood vaccinations~~ in Mysore~~. I~~Future i~~nterventions ~~that aim to improve full vaccination rates in the region may need to work on increasing~~are needed to improve parental awareness about the benefits of childhood vaccination particularly among u~~. The interventions should particularly target u~~neducated parents in rural areas~~ to achieve maximum results~~. There may be other ~~However, lack of significant relationship between parental attitudes about barriers to routine vaccination, and the rate of full vaccination suggests the presence of other additional~~ supply-related factors that require structural intervention to ensure that information and immunization are more widely available, particularly in rural areas~~, which could potentially contribute to the low rates in Mysore~~. Future studies ~~should examine~~on ~~supply~~ supply-related factors ~~t~~are needed~~hat can affect routine vaccination to design appropriate policies for improving the rate of routine vaccination in the region~~. ~~In addition,~~ T~~t~~he current study confirmed complementary relationships among sociodemographic, environment and attitudinal factors in predicting childhood vaccination based on ~~to explain factors that affect routine vaccination as suggested by the~~ HBT. Add~~T. However, ad~~ditional longitudinal studies are needed to confirm these relationships ~~required to examine the causal influence of sociodemographic factors on parental attitudes about routine vaccines, and rate of vaccination to fully verify whether the HBT is applicable to guide childhood vaccination studies in India.~~

Acknowledgements

This study was funded by an ~~Investigator-~~Investigator-Initiated Award from Merck & Co., Inc. awarded to ~~Dr.~~ PM~~urnima Madhivanan~~. KK was funded as a US Scholar by Fogarty International Center and National Heart Lung and Blood Institute, and National Institute of Neurological Disorders and Stroke and of the National Institutes of Health under Award Number D43 TW010540. PM was partially funded by grants from National Institutes for Health (D43-TW010540; R15-AI128714). The funder~~s~~ had no role in the study design, data collection, analysis, interpretation or publication of the manuscript. We would like to thank the study participants for taking the time to complete the questionnaires. We would like to thank also the Block Development Officer for Mysore and the administrative staff of the schools for their assistance during data collection.

Conflict of interest

The authors reported no conflict of interest. The funder does not alter our adherence to PLOS ONE policies on sharing data and materials

Formatted: Font: (Default) Times New Roman, 12 pt

Formatted: Normal, Space Before: 0 pt, After: 0 pt, Pattern: Clear

Formatted: Font: (Default) Times New Roman, 12 pt

References

1. WHO: Immunization coverage. https://www.who.int/news-room/fact-sheets/detail/immunization-coverage. Accessed September 29, 2019.
2. Keja K, Chan C, Hayden G, Henderson RH: Expanded programme on immunization. https://www ncbi nlm nih.gov/pubmed/3176515. World Health Stat Q. 1988;41(2):59-63.
3. WHO: Immunization, Vaccines and Biologicals. https://www.who.int/immunization/newsroom/press/immunization_coverage_july2014/en/. Accessed September 29, 2019.
4. UN: Inter-agency Group for Child. Levels & Trends in Child Mortality. 2013
5. Madhavi Y: Vaccine policy in India. PLoS Med. 2005; 2 (5): e127.
6. Oldfield BJ, Stewart RW: Common Misconceptions, Advancements, and Updates in Pediatric Vaccination Administration. South Med J. 2016;109(1):38-41.
7. Gurnani V, Haldar P, Aggarwal MK, et al: Improving vaccination coverage in India: lessons from Intensified Mission Indradhanush, a cross-sectoral systems strengthening strategy. BMJ., 2018;363.
8. Laxminarayan R, Ganguly NK: India's vaccine deficit: why more than half of Indian children are not fully immunized, and what can—and should—be done. Health Aff. (Millwood), 2011;30:1096–103.
9. Singh PK: Trends in child immunization across geographical regions in India: focus on urban-rural and gender differentials. PLoS One. 2013; 8(9):e73102.

10. Francis MR, Nohynek H, Larson H, et al: Factors associated with routine childhood vaccine uptake and reasons for non-vaccination in India: 1998-2008. Vaccine. 2018;36(44):6559-6566.
11. Choudhary M, Solomon R, Awale J, et al: Demand-side determinants of timely vaccination of oral polio vaccine in social mobilization network areas of CORE Group polio project in Uttar Pradesh, India. BMC Infect Dis. 2018;18(1):222.
12. Shrivastwa N, Gillespie BW, Kolenic GE, et al: Predictors of vaccination in India for children aged 12-36 months. Vaccine. 2015;33:D99-105.
13. Som S~~1~~, Pal M, Chakrabarty S, Bharati P: Socioeconomic impact on child immunization in the districts of West Bengal, India. Singapore Med J. 2010;51(5):406-12.
14. Shrivastwa N, Wagner AL, Boulton M:~~.~~ Analysis of State-Specific Differences in Childhood Vaccination Coverage in Rural India. Vaccines. (Basel) 2019; 24:7(1)
15. Rammohan A, Awofeso N: District-level variations in childhood immunizations in India: the role of socio-economic factors and health infrastructure. Soc Sci Med. 2015; 145:163–172.
16. Rathi A, Meena G: Demand and supply side factors affecting utilization of immunization Services in an Urban Village of Delhi. Adv Tech Biol Med. 2017;5:1.
17. Devasenapathy N, Jerath SG, Sharma S, et al: Determinants of childhood immunization coverage in urban poor settlements of Delhi, India: a cross-sectional study. BMJ Open. 2016;6:e013015.
18. Awasthi A, Pandey CMM, Singh U, et al: Maternal determinants of immunization status of children aged 12–23 months in urban slums of Varanasi, India. Clin Epidemiol Glob Heal. 2015; 3:110–116.

19. Ghosh A, Laxminarayan: Demand- and supply-side determinants of diphtheria-pertussis-tetanus non vaccination and dropout in rural India. Vaccine. 2017;35:1087–93.

20. Gupta P, Prakash D, Srivastava JP: Determinants of immunization coverage in Lucknow district. N Am J Med Sci. 2015; 7:36–40.

21. Kumar S, Prabhu S, Jose AP, et al: Assessment of under-five immunization coverage among the population of slum areas in Mangalore taluk, India. Int J Community Med Public Heal. 2017;4:781.

22. Singhal G, Mathur H, Dixit M, et al: Factors affecting immunization among children of rural population of block Malpura, district Tonk, Rajasthan, India. Int J Community Med Public Heal. 2016;3:641–6.

23. Srivastava AK, Shankar G:. A study of immunization coverage and its determinants among under five children residing in urban field practice area of S. N. Medical college, Bagalkot, Karnataka, India. Indian J Forensic Community Med. 2017;4:48–52.

24. Mukherjee S, Madhivanan P, Li T, et al: Correlates of completing routine vaccination among children in Mysore, India. J Infect Public Health. 2015;8:62–71.

~~25.1. Montaño DE, Kasprzyk D: Theory of reasoned action, theory of planned behavior, and the integrated behavioral model. In: Glanz K, Rimer BK, Viswanath K (Eds.), Health behavior and health education: theory, research, and practice, 4th ed. San Francisco: Jossey-Bass. 2008; p. 67-96.~~

~~26.1. Jones CL, Jensen JD, Scherr CL, et al: The Health Belief Model as an explanatory framework in communication research: exploring parallel, serial, and moderated mediation. Health Commun. 2015;30(6):566-76.~~

Formatted: Font color: Auto

~~27.~~25. Eberhardt MS, Pamuk ER: The importance of place of residence: examining health in rural and non-rural areas. Am J Public Health. 2004; 94 (10): 1682–1686.

26. Das D, Pathak M: The growing rural-urban disparity in India: some issues. Int J Adv Res Tech. (2012): 1-7.

27. Montaño DE, Kasprzyk D: Theory of reasoned action, theory of planned behavior, and the integrated behavioral model. In: Glanz K, Rimer BK, Viswanath K (Eds.), Health behavior and health education: theory, research, and practice. 4th ed. San Francisco: Jossey-Bass. 2008. p. 67-96.

~~28.~~ Jones CL, Jensen JD, Scherr CL, et al: The Health Belief Model as an explanatory framework in communication research: exploring parallel, serial, and moderated mediation. Health Commun. 2015;30(6):566-76.

28.

29. Coe AB, Gatewood SBS, Moczygemba LR, Goode JKR, Beckner JO: The use of the health belief model to assess predictors of intent to receive the novel (2009) H1N1 influenza vaccine. Innov Pharm. 2012:3(2):1-11.

30. Cheney MK, Robert John R: Underutilization of Influenza Vaccine: A Test of the Health Belief Model. SAGE Open; 2013: 1–12.

~~29.~~31. Gerend MA, Shepherd JE: Predicting human papillomavirus vaccine uptake in young adult women: Comparing the Health Belief Model and Theory of Planned Behavior. Ann Behav Med. 2012; 44(2): 171-180.

~~30.~~32. Census; 2011. http://censusindia.gov.in/2011-Common/CensusData2011.html. Accessed Sept 30, 2019.

Formatted: Font: (Default) Times New Roman, 12 pt, Font color: Text 1

~~31.~~33. Degarege A, Krupp K, Fennie K, et al: An integrative behavior theory derived model to assess factors affecting HPV vaccine acceptance using structural equation modeling. Vaccine. 2019;37(7):945-955.

~~32.~~34. Degarege A, Krupp K, Fennie K, et al.: Urban-Rural Inequities in the Parental Attitudes and Beliefs Towards Human Papillomavirus Infection, Cervical Cancer, and Human Papillomavirus Vaccine in Mysore, India. J Pediatr Adolesc Gynecol. 2018;31(5):494-502.

~~33.~~35. Degarege A, Krupp K, Fennie K, et al.: HPV vaccine acceptability among parents of adolescent girls in a rural area Mysore, India. J Pediatr Adolesc Gynecol. 2018;31(6):583-591.

36. Muthén LK, Muthén BO: Mplus User’s Guide. Eighth Edition (1998-2017). Los Angeles, CA: Muthén and Muthén.

~~34.~~37. Hair JF, Tatham RL, Anderson RE, Black W: Multivariate data analysis. (Fifth Ed.) 1998 Prentice-Hall:London.

~~35.~~38. Hu L, Bentler PM: Cutoff criteria for fit indexes in covariance structure analysis: Conventional criteria versus new alternatives. Structural Equation Modeling. 1999; 6(1): 1-55.

~~36.~~39. Byrne BM: Structural Equation Modeling with Mplus: Basic Concepts, Applications and Programming. 2011. Routledge Academic, New York.

~~37.~~40. Muthe´n BO, du Toit SHC, Spisic D: Robust inference using weighted least squares and quadratic estimating equations in latent variable modeling with categorical

and continuous outcomes. Retrieved from www.statmodel.com/bmuthen/articles/Article_075.pdf [Accessed April 2018].

~~38.~~41. Tenko Raykov T, Marcoulides GA: Using the Delta Method for Approximate Interval Estimation of Parameter Functions in SEM. Struct Equ Model. 2004; 11:4, 621-637.

42. Prusty RK, Kumar A:~~.~~ Socioeconomic dynamics of gender disparity in childhood immunization in India, 1992-2006. PLoS One. 2014;9(8):e104598.

~~39.~~43. Ministry of Health and Family Welfare. Indian fact sheet: National Family Health Survey (NFHS-4) Accessed from http://rchiips.org/NFHS/pdf/NFHS4/India.pdf in July 6 2020.

~~40.~~44. Prislin R, Dyer JA, Blakely CH, et al.: Immunization status and sociodemographic characteristics: the mediating role of beliefs, attitudes, and perceived control. Am J Public Health. 1998;88(12):1821-6.

~~41.~~45. Rainey JJ, Watkins M, Ryman TK, et al.: Reasons related to non-vaccination and under-vaccination of children in low and middle income countries: findings from a systematic review of the published literature, 1999-2009. Vaccine. 2011;29(46):8215-21.

46. Vonasek BJ, Bajunirwe F, Jacobson LE, et al:~~.~~ Do Maternal Knowledge and Attitudes towards Childhood Immunizations in Rural Uganda Correlate with Complete Childhood Vaccination? PLoS One. 2016;11(2):e0150131.

47. Skea ZC, Entwistle VA, Watt I, Russell E: Avoiding harm to others' considerations in relation to parental measles, mumps and rubella (MMR) vaccination discussions–an analysis of an online chat forum. Soc Sci Med. 2008;67(9):1382–90.

~~42.~~48. Chaudry A, Henly J, Meyers M: Conceptual Frameworks for Child Care Decision-Making. White Paper. Administration for Children & Families 2010. Accessed from https://www.acf.hhs.gov/sites/default/files/opre/conceptual_frameworks.pdf, on July 7, 2020.

~~43.~~49. Holipah, Maharani A, Kuroda Y: Determinants of immunization status among 12- to 23-month-old children in Indonesia (2008-2013): a multilevel analysis. BMC Public Health. 2018;18(1):288.

~~44.~~50. Khan J, Shil A, Prakash R:~~.~~ Exploring the spatial heterogeneity in different doses of vaccination coverage in India. PLoS One. 2018;13(11):e0207209.

~~45.~~51. Prislin R, Dyer JA, Blakely CH, et al: Immunization status and sociodemographic characteristics: the mediating role of beliefs, attitudes, and perceived control. Am J Public Health. 1998; 88(12): 1821–1826.

52. ~~Patel TA, Pandit NB: Why infants miss vaccination during routine immunization sessions? Study in a rural area of Anand district, Gujarat. Indian J Public Health. 2011;55(4):321.~~Prusty RK, Kumar A: Socioeconomic Dynamics of Gender Disparity in Childhood Immunization in India, 1992–2006. Plos One 2014; 9(8): e104598

~~46.~~ Corsi DJ, Bassani DG, Kumar R, et al: Gender Inequity and Age-Appropriate Immunization Coverage in India From 1992 to 2006. BMC Int Health Hum Rights. 2009;9 (Suppl 1):S3.

53.

Formatted: Font: (Default) Times New Roman, 12 pt

Formatted: List Paragraph, Numbered + Level: 1 + Numbering Style: 1, 2, 3, … + Start at: 1 + Alignment: Left + Aligned at: 0.25 + Indent at: 0.5 , Tab stops: 0.38 , Left + 0.63 , Left

Formatted: List Paragraph, Line spacing: single

Formatted: List Paragraph, Line spacing: Double, Numbered + Level: 1 + Numbering Style: 1, 2, 3, … + Start at: 1 + Alignment: Left + Aligned at: 0.25 + Indent at: 0.5 , Tab stops: 0.38 , Left + 0.63 , Left + Not at 4.45

PONE-D-20-09473
Structural equation modeling to detect correlates of childhood vaccination: a moderated mediation analysis
PLOS ONE

Dear Dr. William Joe

Thank you for your comments and for providing us with the reviewers' comments as well. We thank also the reviewers for their careful review and constructive comments, which have helped to improve the manuscript. We have made changes to the manuscript based on reviewers' suggestions and describe these changes in the below paragraphs. We hope that you will find our responses acceptable and we look forward to your decision.

Editors

1. Please ensure that your manuscript meets PLOS ONE's style requirements, including those for file naming. The PLOS ONE style templates can be found at
https://journals.plos.org/plosone/s/file?id=wjVg/PLOSOne_formatting_sample_main_body.pdf
https://journals.plos.org/plosone/s/file?id=ba62/PLOSOne_formatting_sample_title_authors_affiliations.pdf

Response: We have checked the author guidelines for formatting of the manuscript and to assure the editor that it meets the requirements.

2. Please address the following:
- Please include additional information regarding the survey or questionnaire used in the study and ensure that you have provided sufficient details that others could replicate the analyses. For instance, if you developed a questionnaire as part of this study and it is not under a copyright more restrictive than CC-BY, please include a copy, in both the original language and English, as Supporting Information.

Response: We thank the editor for this suggestion. We have provided sufficient detail about the questionnaire used in this study that would enable others to replicate the analyses. In the manuscript, we have provided all the items and scales/response categories used to measure the outcome (childhood vaccination status) and exposure variables, including the latent (perceived benefits and perceived barriers) and measured variables (sociodemographic variables).

- Please refer to any sample size calculations performed prior to participant recruitment. If these were not performed please justify the reasons. Please refer to our statistical reporting guidelines for assistance (https://journals.plos.org/plosone/s/submission-guidelines.#loc-statistical-reporting).

Response: This study was part of a survey implemented to examine HPV vaccine acceptance among parents of adolescent girls in India. The primary goal of the project was to examine factors associated with intention-to-vaccinate daughters with HPV vaccine. Assessing factors correlated with childhood vaccination was a secondary objective of the project. Therefore, the estimated sample size (n=1599) had enough power to test the hypotheses and answer the research question proposed related to both the objectives of the study.

3.Thank you for stating the following in the Financial Disclosure section:

'This study was funded by an Investigator Initiated Award from Merck & Co., Inc. PM received the fund (D43 TW010540). The funders had no role in study design, data collection and analysis, decision to publish, or preparation of the manuscript.'

We note that you received funding from a commercial source: Merck & Co., Inc.

a. Please provide an amended Competing Interests Statement that explicitly states this commercial funder, along with any other relevant declarations relating to employment, consultancy, patents, products in development, marketed products, etc.

Within this Competing Interests Statement, please confirm that this does not alter your adherence to all PLOS ONE policies on sharing data and materials by including the following statement: "This does not alter our adherence to PLOS ONE policies on sharing data and

materials." (as detailed online in our guide for authors http://journals.plos.org/plosone/s/competing-interests). If there are restrictions on sharing of data and/or materials, please state these. Please note that we cannot proceed with consideration of your article until this information has been declared.

Response: We have provided the text below in the manuscript under the heading "funding"

"This study was funded by an Investigator-Initiated Award from Merck & Co., Inc. awarded to PM. KK was funded as a US Scholar by Fogarty International Center and National Heart Lung and Blood Institute, and National Institute of Neurological Disorders and Stroke and of the National Institutes of Health under Award Number D43 TW010540. PM was partially funded by grants from National Institutes for Health (D43-TW010540; R15-AI128714). The funder had no role in the study design, data collection, analysis, interpretation or publication of the manuscript."

We have also included the statement which reads, "This does not alter our adherence to PLOS ONE policies on sharing data and materials." under the heading "competing interest statement" and in the cover letter. However, I didn't see the financial disclosure and competing statements in the online submission and have not updated the text there. I would greatly appreciate if you can copy this text to the online submission as needed.

Thanks

b. Please include your amended Competing Interests Statement within your cover letter. We will change the online submission form on your behalf.

Please know it is PLOS ONE policy for corresponding authors to declare, on behalf of all authors, all potential competing interests for the purposes of transparency. PLOS defines a competing interest as anything that interferes with, or could reasonably be perceived as interfering with, the full and objective presentation, peer review, editorial decision-making, or publication of research or non-research articles submitted to one of the journals. Competing interests can be

financial or non-financial, professional, or personal. Competing interests can arise in relationship to an organization or another person. Please follow this link to our website for more details on competing interests: http://journals.plos.org/plosone/s/competing-interests

Response: We have included the amended Competing Interests Statement above in '3a' in the cover letter.

Reviewers' comments:

Reviewer #1:

1. In Abstract section, method section needs to rewrite scientifically. Does the children was sample or parents of the children.

Response: We have revised the method section in the abstract to clarify the sampling method. The parent study used a two stage sampling by first selecting the schools. Then a random sample of school students were selected in the age category that was needed for the study and subsequently we reached out to the parents of the selected students. We have not provided details of the sampling procedure and the questionnaire in the abstract due to word limits by the journal. The revised methods in the abstract reads as (line 26-32):

" A cross-sectional survey was conducted among a random sample of 1599 parents living in urban and rural areas of Mysore district, India. Applying two-stage probability proportionate-to-size sampling, adolescent girls attending 7th through 10th grades in public, private and religious schools located in the district were selected to take home a questionnaire to their parents. Parents were asked whether their children had received one dose of bacillus Calmette–Guérin; three doses of Diphtheria, Pertussis, Tetanus; three doses of oral polio vaccine; and one dose of measles-containing vaccine. In addition, parents were asked about their attitudes towards childhood vaccination."

2. In the introduction section, from line number 86 to 92 "Therefore, it is imperative to…………………… immunization rates" seems the aim and objective of the study needs to be placed at the end of the introduction section.

Response: We have rephrased the text "Therefore, it is imperative to……………………. immunization rates" and moved it to the last paragraph in the introduction before the text about the objectives of the study (line 120-122).

The revised text reads as "Knowing sociodemographic, environmental and psychosocial correlates of childhood vaccination will inform the choice of interventions to reduce parental hesitancy to childhood vaccination. The objectives of this study were …….for analyzing childhood vaccination data."

3. As the study intended to examine health belief theory controlled and regulated on individual compositional and contextual factors, it is highly appreciated if authors can write it separately under the section "Health Belief Theory and Immunization Practices". Line no 101 to 112. use no vaccination not unvaccinated.

Response: we have added a paragraph summarizing information about use of Health Belief Theory and Immunization practices. It reads as follows (line 107-119):

"HBT has been used to explain the predictors of vaccination uptake related to influenza, HPV, measles, pneumonia, and meningitis in different populations [29-31]. According to the HBT, six factors including perceived susceptibility to disease, perceived severity of a disease, expected benefits of immunization, concerns or cost associated with immunization, strategies or information sources/media that promote immunization and confidence to adopt/accept immunization would likely affect the chance of getting a vaccine [28]. Children will likely be vaccinated if parents perceive that their children are susceptible to severe diseases, believe that the benefit their children receive through immunization (e.g., reduce susceptibility to severe diseases) outweighs the cost/concerns about the vaccine, and children appear open to receiving the vaccination [28]. HBT has however, been mostly used as a framework for explaining vaccination in Western countries. In addition, the theory lacks the socio-cultural factors which could affect individuals' beliefs and behaviors related to immunization. There remains limited information on the utility of the HBT to explain parental attitudes and beliefs about childhood vaccination in developing countries like India."

We have also replaced unvaccinated with 'no vaccination' throughout the manuscript (line 200-206 and line 195 (Table 2).

4. Line no 112 onwards is existing gap and support for your argument to examine HBT framework in Indian scenario followed by objectives. So, the line number 86 to 92 will also be part of this section.

Response: we have rephrased the text from line number 86 to 92 in the older version of the manuscript "Therefore, it is imperative to……………………. immunization rates" and moved it to the last paragraph in the introduction before the text about the objectives of the study. We used the text to support the rationale for the study.

5. Overall, Introduction section needed to rewrite with proper flow setting, as the line no 123-124 should not be part of this section.

Response: We have made major revisions in the introduction following your suggestions above and below. Please see comment 15 below for additional revisions we have made in the introduction. **(line 120-122).**

6. In the Materials and Method section, Study design needs clarification. As the authors have selected adolescent girl children. In Indian scenario, gender dimension works differently. For the purpose authors are advised to go through gender biasness in immunization in India. As within demographic and health studies in developing counties including India have found disparity, discrimination and exclusion regulated by socio-economic and cultural factors contribute majorly in resource allocation and achievement which lead to poor outcome for female (child, adolescent and adult). Had the authors all these issues in mind when they were finalizing the sampling frame. So within the HBT framework, gender dimension needs to be included. Even it has not discussed in HBT framework section needs attention.

Response: We have revised the study design and placed it under the heading 'Study design and participants recruitment procedure'. It reads as follows:

"A cross-sectional study was conducted among a random sample of parents whose daughters aged 11 to 15 years were attending 7th through 10th grades in schools located in the urban and rural areas of Mysore District. In order to increase the chance of inclusion of parents with a

variety of backgrounds while ensuring representativeness of the samples, two-stage probability proportionate-to-size sampling was applied to select adolescent girls attending public, private and religious schools located in the district.

Study participants recruitment procedures are explained in detail elsewhere [33,34]. In brief, 12 schools in the urban area (five government, four private and three religious) and 11 schools in the rural areas (10 government and one private) of Mysore District were selected. A program announcement was sent home with all girls in 7th through 10th grades in the selected schools, explaining the objectives of the study and inviting eligible parents to participate. Of all the girl students in the selected age group in the select schools, 800 girls from urban schools, and 850 girls from rural schools were randomly selected and provided with a questionnaire and consent form to take home to their parents. The girls were expected to return the completed questionnaire and signed consent form within a week. Only one parent was required to complete the questionnaire. A small proportion of parents in the urban (2.7%) and rural (2.2%) areas did not return the completed questionnaire and/or the sign the consent form. IRB approval for this study was obtained from Florida International University and Public Health Research Institute of India."

We have also discussed the limitations of the generalizability of the current finding due to potential gender bias in immunization in India. It reads as follows (Line 428-431):

"Finally, we are reporting on data collected from parents who had adolescent daughters attending school during the study period. As a result, the generalizability of the current results might be limited to parents with at least one school-going adolescent daughter. The generalizability of the study findings to parents without school-going adolescent girls or with only school-going boys will be further limited due to gender biases in immunization in India [52,53]."

7. Further, the question is why the authors had selected female children of age 11-15 years, if they were interested in examining the regulators and predictors of basic immunization in Mysore. As per the guidelines, the basic immunization what the authors have mentioned can be completed within the age of one year as the measles vaccine is given at last after completing the age of 9 months. So, question arises that perception about vaccination may have altered over the period of 10-14 years due to various factors. So, how this HBT can be applied, is another concern.

Response: This was a secondary analysis of data from a study implemented to examine HPV vaccine acceptance of parents in India. The primary goal of the project was to examine factors affecting intention to accept HPV vaccine for the girls as the HPV vaccine is only licensed for them in India. Assessing factors correlated with childhood vaccination was a secondary objective of the project. As the Indian government approved HPV vaccination to only adolescent girls during the study period, we targeted parents who have at least one adolescent daughter during the study period. To reach out to these parents effectively, we selected adolescent girls attending schools during the study period. However, we acknowledge that since the study participant was a parent of atleast one adolescent daughter, there is a potential for selection bias and information bias as we are asking them about events that took place more than ten years ago, and this may limit the applicability of the HBT to explain factors predicting childhood vaccination in the Indian population. We have discussed this as a limitation of the study. It reads as follows (line 428-437): "Finally, we are reporting on data collected from parents who had adolescent daughters attending school during the study period. As a result, the generalizability of the current results might be limited to parents with at least one school-going adolescent daughter. The generalizability of the study findings to parents without school-going adolescent girls or with only school-going boys will be further limited due by gender biases in immunization in India [52,53]. It is also possible that there may be recall bias and parental attitudes may have changed about childhood vaccinations. Thus, policy measures designed to change negative attitudes and beliefs of parents about childhood vaccination based on the current results should be done carefully considering potential changes over the period of time."

8. Further, are all the selected schools government or private? As the affordability to cater and send the children to these two type of school matters more. It is also important to add here that there is high possibility of very few private schools in rural areas. So, the authors have to mention the selection criteria for school is also needed.

Response: In order to include parents with a wide range of background and increase the representativeness of the sample to the population in the study area, we have included parents of adolescents who were attending government, private and religious schools. The schools were selected randomly using proportionate to size. The revised text about the type of schools selected and the selection procedure reads as follows: (line 138-145)

"In order to increase the chance of inclusion of parents with a variety of backgrounds while ensuring representativeness of the samples, two-stage probability proportionate-to-size sampling was applied to select adolescent girls attending public, private and religious schools located in the district. Study participants recruitment procedures are explained in detail elsewhere [33,34]. In brief, 12 schools in the urban area (five government, four private and three religious) and 11 schools in the rural areas (10 government and one private) of Mysore district were selected based on probability proportionate to size sampling."."

9. Authors have mentioned about majority is living in rural areas. As per the 2011 census, close to 69% of country population and 61% of state population was living in rural areas. In case of Mysore, it is close to 58 percent. So using majority for Mysore district is not appropriate here.

Response: We thank the reviewer for this comment and have replaced majority with 58.5%. The revised text reads as: "Of the 3,001,127 people living in the district (density = 450/km2), 58.5% live in rural areas (1,755,714)". (line 133, 134)

10. In the text author have mentioned four categories for each of the facilitators and barriers for the child vaccination but in the table option, only three categories is provided. The table can be in appendix section.

Response: We have corrected the response categories text in the table. We have removed the column with response categories in the table and provided that same information as a footnote in the table. It reads as follows: 'Responses to all the items were recorded in four categories: 0= No, 1= Not sure, 2= Yes, 3= Not applicable."

11. In the line number 142 "Only one parent in each family was required to complete the questionnaire". It seems fine but In Indian scenario, how is it possible that 112 are unmarried in table 2 who are having girl child/children of age 11-15 years. Are they male or female?. As per the knowledge and understanding, In India, without marriage, giving birth was not acceptable. However, there may be possibilities that if the parent are unmarried, they would have adopted it or else. So, authors have to clearly mention about who are those single parent.

Response: We thank the reviewer for pointing out this statement. We want to clarify that we have included the definition for 'single parent' in Table 2 as a footnote. It now reads as "Single=unmarried or separated or widowed". Most of the parents who reported their marital status as single were separated or widowed.

12. Line No 191-202 is contextualizing and purpose of use of SEM to model and examine the magnitude and direction of facilitator and barrier predictors controlled on Parent SES characteristics, seems fine but need to rewrite in scientific manner. In the next section, it seems human error has happened as sign of 'greater than' and 'lesser than' is incorrect.

Response : We thank the reviewer for catching this error. We have revised the text about the SEM and have corrected the sign of 'greater than' and 'less than in the data analysis on the method section. It reads as follows (line 227-246):

"SEM was used to examine parental perceptions that directly predicted childhood vaccination, and sociodemographic factors that indirectly predicted the behavior. SEM was also used to evaluate the mediating roles of parental perceptions on the relationship between sociodemographic factors and childhood vaccination and the moderating role of 'rural or urban residence' on the relationship between sociodemographic factors and parental perceptions about childhood vaccination. Moreover, SEM was applied to test model fit of the proposed HBT derived model (Fig 1) with the observed data.

Model fit was checked using chi-square (model acceptable if $P > 0.05$) and other fit indices including Comparative Fit Index (model acceptable if CFI > 0.90), Tucker–Lewis Index (model acceptable if TLI > 0.90), and Root Mean Square Error of Approximation (model acceptable if RMSEA is < 0.08) [38]. Before fitting the SEM model that has the measurement and structural components in it, fit of the measurement model that included the constructs' perceived benefits/facilitators' and 'barriers' to childhood vaccination was checked. In order to improve the fit of the measurement model, a slight modification was made by removing items with factor loading < 2 and by allowing some of the items to freely co-vary. Similarly, we introduced slight changes in the SEM model based on the Mplus model fit indices output [39]. Since the outcome variable (full vaccination status) was categorical in nature, the Weighted Least Squares Estimation Method was used to estimate the parameters (path coefficients, factor loadings,

variances, and covariances) and model fit statistics [40]. Standard errors and 95% CI for direct and indirect effects were estimated using the delta method [41]."

13. Measurement model and structural model can be single section and need methodological and scientific rigor and elaboration.

Response: We have merged the text about the measurement and structural model under a single heading (Structural equation model) and revised the text to better explain the model. The revised text about the mode reads as follows (line 259-307):

Structural Equation Model

"The measurement model for the construct 'perceived benefits/facilitators to childhood vaccination' that included all the items listed in table 1 fitted acceptably to the data based on the RMSEA. But the model showed lack of fit based on CFI and TLI values (RMSEA = 0.05, CFI = 0.89, TLI = 0.86). After allowing some residual terms for items to covary (F1 WITH F2; F3 WITH F4; F10 WITH F11; F10 WITH F12; F11 WITH F12), fit of the construct measurement model 'perceived benefits/facilitators to childhood vaccination' improved (RMSEA =0.03, CFI =0.97, TLI =0.96). All 12 items listed in Table 1 were used to measure the construct 'perceived benefits/facilitators' that significantly loaded with standardized factor loadings (β) ranging from 0.27 to 0.75. The items "It is very important that my children receive all their vaccine" and "I have a responsibility to have my children vaccinated for the protection of all children" explained sufficient variance or strongly influenced/correlated (β = 0.75 and 0.73, respectively), and the item "Parents should make health decision for their own children rather than a doctor" explained small variance weakly influenced/correlated (β = 0.27) with the score for the construct variable 'perceived benefits/facilitator' to childhood vaccination. The remaining nine items moderately influenced/correlated with the score of the construct variable perceived benefits/facilitator (β = 0.43 to 0.67).

The measurement model for the construct 'perceived barriers to childhood vaccination' fitted acceptably to the data (RMSEA = 0.06, CFI = 0.94, TLI = 0.92, β = 0.12 to 0.66), but the factor loading index for the item 'I would feel responsible, if anything bad happened I had my child vaccinated' was 0.12. After removing items with a factor loading less than two, and allowing the residual terms for some items to covary (B5 WITH B6; B6 WITH B8), data fit of the measurement model for the construct 'barriers' to childhood vaccination improved (RMSEA =

0.05, CFI =0.98, TLI =0.96). Seven items, B1- B3 & B5 - B8 used to measure the construct 'perceived barriers' significantly loaded with a standardized factor loading ranging from 0.20 to 0.73. The item "Getting time off from work or household duties makes it difficult to take my child for vaccination" explained sufficient variance or strongly influenced/correlated ($\beta = 0.73$) and the item "I am concerned about vaccine side effects" explained small variance or weakly influenced/correlated ($\beta = 0.20$) with the score for the construct variable perceived barriers to childhood vaccination. The observed scores for the remaining four items moderately influenced/correlated with the score of the construct variable perceived benefits/facilitator to childhood vaccination ($\beta = 0.43$ to 0.67). The item "It is better to get the disease and protected" showed a modest-sized positive influence/correlation ($\beta = 0.35$) with the construct variable perceived benefits/facilitator to childhood vaccination. The final measurement model that included 19 items used to measure facilitators and barriers to childhood vaccination, had acceptable fit the data. (RMSEA =0.04, 95% CI: 0.03 - 0.04; CFI =0.93, TLI =0.91) (Fig 2).

Fig 2. Measurement model: factor loadings of items used to measure facilitators and barriers about childhood vaccination in Mysore, India 2010/2011.

The final full SEM model with the structural and measurement components was identified. The total number of free parameters estimated (#130) was less than the number of model parameters (#630). The number of free parameters was the sum of the numbers of estimates, including factor loadings, variances of error and covariance. The number of model parameters is the number of unique covariances of measured variables estimated as the product of the number of measured variables (#35) (the number of measured variables +1) divided by 2.

Fit of the final SEM model to the data was also acceptable based on RMSEA, CFI and TLI values (RMSEA: 0.02: 90% CI: 0.018 to 0.023, CFI=0.92; TLI=0.91) (Fig 3). The chi-square statistic however, suggested a significant difference in the covariance matrix in the proposed model (Fig 1) and the observed data ($\chi 2 = 3914.6$, DF=490, $p < 0.01$).

Fig 3. Structural Equation Model showing moderators, mediators and direct and indirect predictors of childhood vaccination in Mysore, India, 2010/2011."

14. It is also observed that when author is using coefficient value, used "=" sign for p values, needs correction at many places.

Response: We have corrected use of the = sign (added space) and 'p' (capitalized, italicized and added space) in the revised manuscript.

15. As the author used SEM to depict facilitator and barriers in child vaccination moderated by parents SES suing primary data collected from Mysore, authors should add why it is more appropriate in the study as other methods like path analysis, simultaneous equation modelling and CFA etc.

Response: We have revised the text in the introduction to give readers why SEM is a more appropriate method to model predictors of childhood vaccination and explain the relationship between the predictors effectively as compared to other methods including logistic regression analysis, path analysis, simultaneous equation modelling and CFA. The revised text reads as follows (Line 84-107):

"Most of these studies however assumed direct relationships between sociodemographic, environmental, psychological factors and childhood vaccination in logistic regression models [12-26]. On the other hand, some predictors of childhood vaccination may have a direct effect, and some may have an indirect effect. Furthermore, some may play a mediating or moderating role between the predictors and childhood vaccination [27,28].

Evidence based on Health Belief Theory (HBT) suggests that factors that affect preventive behaviors such as vaccination are complex and multifaceted, and do not always act in similar manners [27,28]. According to HBT, individual perceptions about the benefits and barriers to behavior would directly affect the practice of behavior, the effect of sociodemographic status on behavior on the other hand, would be indirect, influencing individual perceptions about the benefits and barriers of behavior [27,28]. In addition, as sociodemographics vary between urban and rural residents in India, [8,9], we hypothesize that the effect of sociodemographic factors may vary by place of residence (urban and rural). Despite the availability of health frameworks that can help us examine factors affecting childhood vaccinations in a comprehensive manner, most studies on childhood vaccination in India did not use a theoretical framework [12-26]. Moreover, some predictors of childhood vaccination can be directly observed, as are some latent variables or constructs [27,28]. As a result, methods including standard logistic regression, factor analysis, simultaneous equation modelling and path analysis may not always be appropriate to model predictors of childhood vaccination, and exploring the relationships between the

predictors. Health theory-driven complex models that employ robust analytic techniques (e.g. Structural Equation Model) are needed to better explain the nature of the relationship between sociodemographic, attitudinal and environmental factors while predicting correlates of routine childhood vaccination."

16. In the discussion section, the statement made needs clarification and updation as well. Line no-313 to line no 320 needs correction. As the sriva statava study is based on third round of DLHS survey data of India conducted during 2007-08, not at three consecutive periods of DLSH-1, DLHS-2 and DLHS-3. Further, as the author is submitting the paper in 2020, the latest available data and report for NFHS is for 2015/16 and it also provide estimates at district level for many indicators including for immunization.

Response: We are grateful for the reviewer for finding this typo. We have revised the results of the study by Shrivastwa, et al. Vaccine. 2015;33:D99-105

The revised text in the discussion reads as follows (line 375-377): "A pooled analysis of 108,057 Indian children aged 12–23 months during a national survey for the period 2007–08 reported similar rates of full childhood vaccination of 65.6% for urban vs 53.6% for rural, residents."

We have also discussed and compared the current finding on full vaccination rate between urban vs rural residents with the Indian National Family Health Survey data collected during 2015–2016 and cited the reference. The revised text in the discussion reads as (line 377-380): "Analysis of the National Family Health Survey data collected during 1992–93, 1998–99, 2005–06 and 2015–2016 also showed a lower vaccination rate among rural Indian children compared to those living in urban areas [9, 42, 43]."

Reference: Ministry of Health and Family Welfare. Indian fact sheet: National Family Health Survey (NFHS-4) Accessed from http://rchiips.org/NFHS/pdf/NFHS4/India.pdf in July 6 2020.

17. There is an impression that study examines the HBT in child immunization behaviour using the data from Mysore district, Karnataka and modelled through SEM, but in the result as well as discussion part, detailed on facilitator and barriers seems missing. Although, Parents SES are discussed with more for education.

Response: We have provided details on facilitators and barriers to childhood vaccination in the results and discussion based on the data in table 3 and fig 3. The revised text in the results reads as follows (line 310-328):

"Full childhood vaccination significantly increased with parental perceptions about the benefits/facilitators to childhood vaccination (standardized regression coefficient (β) = 0.29, P < 0.001). In other words, it increased if parents felt the vaccine was effective in preventing disease and ensuring child health, feeling a responsibility to protect their child and others, having a healthcare provider recommendation, receiving information about childhood vaccine from a doctor or nurse, knowing where to go for vaccinations, perceiving that the government does a good job providing vaccines and health services, and having several vaccines included in the childhood vaccination schedule. Parental education was significantly associated indirectly with increased full vaccination (β = 0.08, P < 0.001). Parental employment was indirectly associated with a decreased rate of full vaccination (β = - 0.05, P = 0.05). The relationship of parental education (β = 0.08, P < 0.001) and employment (β = -0.06, P = 0.045) with the rate of full vaccination were significantly mediated by parental perceptions about the benefits/facilitators to childhood vaccination. Parental perceptions about barriers to childhood vaccination neither showed a significant association directly with the rate of full vaccination nor mediated the relationship between sociodemographic variables including age, gender, marital status, religion, education, occupation and number of children, and the rate of full vaccination (Table 3). In addition, age, gender, marital status, religion and number of children in a family had no indirect relationship with the rate of full vaccination through the construct about perceptions about the benefits/facilitators (Table 3)."

The revised/added text about the influence of perceived benefits/facilitators on childhood vaccination and its implications for intervention in the discussion reads as follows (line 384 to 412):

"Parents perceiving that childhood vaccination was beneficial (e.g. effective in preventing disease, ensure their child health, help protect other children); those who felt responsible if anything bad happened did not have their child vaccinated, and those who heard about the vaccine or received a positive recommendation from a doctor or nurse, were more likely to

report full vaccination. In addition, parental attitudes about childhood vaccination played a mediating role between sociodemographic characteristics and 'full vaccination' rate and this conquered with a previous report [44]. A number of studies in India, Pakistan, Nigeria, Benin and Uganda reported similar results for the relationship between levels of vaccination and parental attitudes and beliefs related to immunization [45,46]. Similarly, a UK study showed that the need to protect children and help protect others through herd immunity, were seen to positively influence parental decisions about vaccinations [47].

These findings indicate key issues in the perceived benefits domain of HBT, that could be leveraged to improve childhood vaccination rates. Community-based education campaigns focused on increasing parental awareness about the benefits of childhood vaccination in preventing disease and promoting child health may be useful. In addition, strategies led by physicians and nurses should focus on strong childhood vaccination recommendations. The psychological decision-making frameworks suggested that when individuals are uncertain, they are more open to information about vaccination in their decision-making [48]. This study also suggests that intervention to increase vaccination in children may target change in parental perception about the benefits of vaccination in protecting others, which agree with the vaccine decision-making framework [47]."

Overall, authors are advised to restructure the paper as per framework mentioned and modelled using SEM. Sampling design written should be valid and justified. Methodology section needs linking with framework and the limitations of the same. Result and discussion section should more focus on facilitator and barriers how regulated by HBT is another concern of the paper. Authors should take care of grammatical errors and editing of the manuscript.

Response: We have followed your suggestions above and made the necessary changes in the abstract, introduction, methods, results and discussion in this revised version of the manuscripts. Hopefully, these changes will address your concerns.

We have also further edited the manuscript for grammar and language.
