# Supplementary material for: Structural equation modeling to detect correlates of childhood vaccination: A moderated mediation analysis
Source: PLoS One. 2020 Oct 15;15(10):e0240749. doi: 10.1371/journal.pone.0240749 (PMC7561155; doi:10.1371/journal.pone.0240749)
Supplement: Attachment — Submitted filename: Response letter.docx [file pone.0240749.s003.docx]

Dear Dr. William Joe

Thank you for providing us with the reviewers' comments. We thank also the reviewers for their careful review and constructive comments, which have helped to improve the manuscript. We have made changes to the manuscript based on reviewers' suggestions and describe these changes in the below paragraphs. We hope that you will find our responses acceptable and we look forward to your decision.

**Reviewer #1:** The authors have taken care of all the comments and suggestions. The manuscript seems improved and revised in logical manner. So, there is not more suggestions other than about the title of the paper.

**Response:** We think that the phrase ‘health belief theory framework’ is superfluous in the title, so we removed it to reduce the verbiage and make the title eye catching.

**Reviewer #2** Please refer to the attached manuscript marked up with my feedback

1. This hyphen should be removed. (Evidence-based)

**Response**: we have revised Evidence-based as Evidence based (line 94)

2. No need to capitalize (Structural Equation Model)

**Response**: we have revised Structural Equation Model as structural equation model (line 109)

3. According to ~~the~~ HBT…..

**Response**: we have removed article ‘the’ in the phrase “According to ~~the~~ HBT” (line 114)

4. Would be interesting to add at least a sentence describing why HBT has been applied disproportionately to Western countries.

**Response**: We don’t think there is any specific reason that HBT has been only applied in Western Countries…Indian studies have not used theory driven approaches as they may not have been aware of such a thing. Most studies in developing countries are conducted by doctors or folks in the medical field and they don’t learn health theories. Still we have added a text that we think may partly be the reason why HBT might been applied mostly in western countries. The revised text reads as “HBT has, however, **been developed to understand predictors of disease prevention behaviors in the United States** and is mostly used as a framework for explaining vaccination in Western countries.” (line 122)

5. Describe why girls specifically?

**Response**: We have provided the reasons why only girls were selected. The added text reads as “This study is a secondary analysis of data collected for a project that examined factors affecting parental intention to accept HPV vaccine for the daughters in Mysore, India [33,34]. Assessing factors correlated with childhood vaccination was a secondary objective of the project. The Indian government has approved HPV vaccination only for adolescent girls [35,36], the parent study; thus, targeted parents who had at least one adolescent daughter during the study period.” (line 140-144).

6. Replace living with reside

**Response:** We have replaced the term living with residing (line 134)

7. Was there a question about the immunization status of the parents as children?

**Response:** there was no question about the immunization status of the parents included in the questionnaire

8. Would briefly restate that these categories were defined consistently with the WHO criteria

**Response:** we have reiterated that the full vaccination definition is based on WHO

The revised text reads as “‘Full vaccination’ described families with children that had received one dose of BCG, three doses of DPT, three doses of OPV and one dose of MCV as recommended by WHO.” (Line 219-221).

9. Please give rationale for choosing the age cutoff at 35 years

**Response**: Age was initially collected as a continuous variable. However, we assumed a better fit of the SEM model when age included as a categorical/binary variable. In addition, the mean age of the study participants was 38.3 with a standard deviation of 6.6.

10. This seems to be a rushed but potentially important point. The authors imply that the size of this group was small and so would be the impact of their responses on the analysis. This is an overreach (even if it is intuitive) because the impact of their responses was not evaluated.

**Response**: We thank the reviewer for picking this. In addition to the size of the response, issues related to the model fit and interpretation of the results were reasons for the exclusion of the ‘not applicable’ category of some items used to collect information on parental attitudes about childhood vaccination. However, we acknowledge that removing this category may have affected the coefficient estimated in the Final SEM model. Thus, we have discussed this as a limitation. It reads as follows “Moreover, treating the ‘not applicable’ response to some items used to assess parental attitudes about childhood vaccination as missing data might have biased the estimated coefficients in the SEM model. However, the proportion of the study participants who responded as ‘not applicable’ were ≤1% to some items that examined parental attitudes about childhood vaccination and even none for some of them. As a result, the effect of the ‘not applicable’ response on the coefficient estimates could be very minimal.” (line 433-438)

The text which reads “Similarly, the number of participants who reported ‘not applicable’ or “not sure” were also small (<10), so they were excluded from the analysis” was a typo and have been removed.

11. Perhaps I'm missing something but how well can one reply to the questionnaire if she/he is illiterate? Further how might literacy have affected response rates or responses? Did the issue of literacy impact the design or interpretation of this study?

**Response**: We acknowledge that illiterate parents might have gotten support from family members or neighbors. And we have included education status that includes illiteracy as a separate category in the model. In addition, we have discussed the potential impact of the illiterate nature of the parents on their response. The added text in the discussion reads as ‘Illiterate parents might have also received support from family members or friends to respond to some questions leading to information bias.” (Line 430,432).

12. **Reiterating here the earlier comment about literacy**

**Response**: We would like to refer the reviewer to our response above.

13. Suggest revising this sentence for clarity

**Response**: We have revised the text which reads ‘In addition, unlike most many studies in India [12-26], did not use as this study did, a health theory-driven model with robust analytical techniques (SEM).” as “In addition, unlike many studies in India [12-26], the current study analyzed data using a health theory-driven model with robust analytical techniques (SEM).” (line 417,418).

14. And simultaneously controlling for parent age correct?

**Response**: Yes, we have controlled for the effect age when we analyzed data after stratifying by the number of children. We have revised the text to reflect this as **“**However, after stratifying data based on the number of children **and simultaneously controlling for age**, full immunization rates remained similar between parents with one child versus those with two or more. (line 427-429).

15. One would expect the overall reported vaccination rates to have been higher if this was the case correct? ***“****Due to the self-reported nature of the data, parents may have been answered questions differently for reasons of social desirability”*

**Response**, Yes, that is true mostly in the urban population but for the rural population may still have a different perspective due to cultural and religious reasons. However, we thought this has more to do with recall than social desirability bias and removed the text “ *Due to the self-reported nature of the data, parents may have been answered questions differently for reasons of social desirability”* from the revised MS.

16. Reiterating here the earlier point about the need to explain more clearly why only girls were chosen

**Response**: We would like to refer the reviewer to our response above.

17. Conclusions: Were any important or interesting outcomes observable or observed with respect to religion? Even if not reliably valuable readers will be looking for some consideration of this topic.

**Response**: We have added a conclusion remark of the finding about the role of religion on parental perception about barriers to vaccination in the conclusion.

The added text reads as “Area of residence may modify the effect of religion and the number of children in a family on parental perceptions about barriers to childhood vaccination.” (line 454 to 456).
